# Analysis of HERV-K (HML2) Expression in Colorectal Cancer Samples

**DOI:** 10.3390/epigenomes10010011

**Published:** 2026-02-12

**Authors:** Valentina S. Obrezanenko, Polina M. Shulga, Anastasia G. Volkova, Anastasia A. Primova, Yulia A. Remizova, Ivan O. Meshkov, Alexandra D. Kikot, Daria A. Tarasova, Ekaterina S. Bolashova, Alexey A. Ivashechkin, Antonida V. Makhotenko, Ekaterina A. Snigir, Yulia A. Masyukova, Elizaveta I. Radion, Olesya A. Kuznetsova, Maria S. Cheporova, Michail Y. Fedyanin, Alexey A. Tryakin, Valentin V. Makarov, Vladimir S. Yudin, Anton A. Keskinov, Anna S. Makarova

**Affiliations:** 1 Federal State Budgetary Institution «Centre for Strategic Planning and Management of Biomedical Health Risks», Federal Medical and Biological Agency (Centre for Strategic Planning, of the Federal Medical and Biological Agency), Moscow 123182, Russia; obrezanenco@mail.ru (V.S.O.); shuulgap@gmail.com (P.M.S.); anastasiiaprimova@gmail.com (A.A.P.); yuremizova@cspfmba.ru (Y.A.R.); imeshkov@cspfmba.ru (I.O.M.); akikot@cspfmba.ru (A.D.K.); datarasova@cspfmba.ru (D.A.T.); ebolashova@cspfmba.ru (E.S.B.); aivashechkin@cspfmba.ru (A.A.I.); amahotenko@cspfmba.ru (A.V.M.); esnigir@cspfmba.ru (E.A.S.); yumasyukova@cspfmba.ru (Y.A.M.); makarov@cspfmba.ru (V.V.M.); vyudin@cspfmba.ru (V.S.Y.); keskinov@cspfmba.ru (A.A.K.); amakarova@cspfmba.ru (A.S.M.); 2 Federal State Budgetary Institution “NN Blokhin National Medical Research Center of Oncology”, Ministry of Health of the Russian Federation (NN Blokhin NMRCO), Moscow 115478, Russia; lessya.kuznetsova@gmail.com (O.A.K.); masha.cheporova@mail.ru (M.S.C.); fedianinmu@mail.ru (M.Y.F.); atryakin@mail.ru (A.A.T.)

**Keywords:** HERV-K (HML-2), colorectal cancer, RNA-seq, DNA methylation, CMS, viral mimicry

## Abstract

Background: HML-2 subgroup mobile genetic elements of the HERV-K family were described to participate in carcinogenesis processes, but their expression and epigenetic regulation in molecular subtypes of colorectal cancer (CRC) remain partly characterized. The present study aimed to evaluate the expression of HML-2 elements using RNA-sequencing data in paired tumor and normal intestinal tissue samples from 63 patients with CRC to identify patterns of the retrotransposons’ activity in different molecular subtypes (CMSs). Methods: RNA-sequencing and DNA methylation data were analyzed for paired CRC and normal tissue samples. HERV-K expression was assessed using three bioinformatics tools: Telescope (version 1.0.3), TEtranscripts (version 2.2.3), GeneTEFlow (version 2020). Molecular tumor subtypes were defined using the CMScaller (version 0.99.2) program. The results of the HML-2 loci expression analysis were supplemented with the HML-2 proteins expression data obtained by quantitative RT-PCR. Results: HML-2 expression assessment by GeneTEFlow (version 2020), TECount (version 2.2.3) and Telescope (version 1.0.3) showed high convergence: the Pearson correlation coefficient for each tool exceeded 0.88. Several HML-2 loci were identified as differentially expressed in CRC samples of different CMS. The PCR results confirmed an increase in HML-2 expression in tumor tissues. For all CMSs, an inverse association was detected between differential methylation of CpG sites and differential expression of HML-2 loci. Associations of HML-2 expressions with differentially expressed genes in which they are located were found, and for a number of such genes an inverse relationship between the expression level and the methylation level of their promoters were demonstrated, and data on the involvement in the pathogenesis of CRC were described: *CR1*, *CD48*, *TTLL3*, *ABCC2* and *ZNF420*. Expression signatures associated with the activity of the RIG-I-like receptor signaling cascade were identified in CMS1–3 CRC samples, which may indicate the possible implementation of viral mimicry against the background of HML-2 activation. Conclusions: Analysis of the expression of HML-2 and its association with CpG methylation contributes to a comprehensive interpretation of the CRC pathogenesis mechanisms.

## 1. Introduction

Colorectal cancer (CRC) is the third leading cause of cancer death and the second most common type of cancer among adults under 50 years of age [1]. In 2022, 1.93 million new cases of CRC and 0.9 million patient deaths due to CRC were detected worldwide [2]. The highest incidence is recorded in Europe, Australia and New Zealand, and the highest mortality is observed in Eastern Europe. According to the forecast of the International Agency for Research on Cancer (IARC), the number of newly diagnosed cases of CRC will increase by 63% and reach 3.2 million per year by 2040, and the number of deaths will be as high as 1.6 million per year (an increase of 73%) [2].

CRC is one of the types of cancer for which effective treatments exist. However, in 20% of cases the disease is detected at the metastatic stage, and the five-year survival rate for such patients does not exceed 20% [3]. The use of methods for identifying biological markers and molecular genetic classification of tumors contributes to early detection and selection of the optimal strategy for treating CRC. Approximately 84% of sporadic cases of CRC are characterized by chromosomal instability: translocations, amplifications, deletions and other types of chromosomal rearrangements. About 16% of sporadic CRC cases are characterized by microsatellite instability (MSI) and accumulation of mutations resulting from deficiency of the DNA mismatch repair (dMMR) system [4]. MSI tumors often exhibit hypermethylation of CpG islands in gene promoters, which leads to the inactivation of tumor suppressor genes, including the MMR system gene *MLH1*. In 2016, a consensus molecular classification of CRC into subtypes (CMSs) based on gene expression profile of the tumor was proposed [5]. The CMS1 subtype accounts for about 14% of cases and is characterized by a high mutation rate, MSI and high levels of immune activation. The 37% of CMS2 CRC cases are characterized by chromosomal instability and Wnt and Myc signaling pathways activation. KRAS mutations are often detected in tumors of CMS3 (13%). Genes involved in epithelial–mesenchymal transition (EMT), angiogenesis and activation of the transforming grow factor β (TGF β) signaling pathway are overexpressed in tumors of CMS4 (24%). Samples with mixed features (13%) possibly represent a transition phenotype or intra-tumoral heterogeneity [5]. It was shown that CMS2 tumors often metastasize to the liver, and CMS4 tumors to the lungs and peritoneum [6]. Tumor location is associated with its molecular genetic characteristics: right-sided tumors are often associated with microsatellite instability, *BRAF* mutations and high levels of CpG island methylation (CIMP-high), compared to left-sided tumors [7].

The tumor microenvironment (TME) of CRC is enriched with regulatory T cells, which downregulate effector T cells present in the TME. This prevents the development of the body’s immune response to tumor antigens [8]. MSI results in the production of large numbers of neoantigens in the tumor. As a result, CMS1 tumors exhibit high levels of the adaptive and innate immune systems leukocyte infiltration [9]. CMS4 tumors are also characterized by pronounced infiltration, but, unlike the CMS1 subtype, the composition of the immune infiltrate is the attribute of the chronic inflammation that occurs in tumors of this type [9].

In addition to the four CMSs, iCMS2 and iCMS3 subtypes were identified based on transcriptome profiling of single tumor epithelial cells [10]. In combination with CMSs 1–4, this generates five subtypes and makes tumor phenotyping more accurate. However, this classification does not exhaust the heterogeneity of CRC. It is possible to further improve and refine the molecular stratification of CRC using a multi-omics approach that combines the analysis of genomic, epigenomic, transcriptomic and proteomic tumor profiles [11].

Human endogenous retroviruses (HERVs) are mobile genomic elements that arose as a result of the integration of ancient exogenous retroviruses into human chromosomal DNA. Integrated chromosomal retroelements in the human genome are usually affected by inactivating mutations, but recently data have been accumulating on the possible influence of such retroelements on the human body [12]. Transcription of HERV was detected in all tissues of the human body without exception, the highest in the skin, placenta and reproductive organs, and the lowest in muscle cells [13]. Phylogenetically, HERVs are divided into several dozen groups. The most recently acquired in our genome and the most studied is the HERV-K group, where “K” stands for lysine, since this group of HERVs uses lysine tRNA for reverse transcription [14]. The HERV-K group is divided into 11 HML (human mouse mammary tumor virus-like) subgroups, HML-1-11. The HML-2 subgroup appeared in the human genome later than the others and therefore is the least defective and most capable of influencing the human body [15]. There is evidence of HERV overexpression in patients with prostate cancer, breast cancer, colorectal cancer [16,17] and other types of cancer [16,18].

To detect polymorphic HERV-K insertions at the DNA level, analysis is performed using whole-genome sequencing (WGS) and specialized computational tools that leverage unique k-mers to identify integration sites specific to individual loci. This approach was implemented in the 1000 Genomes Project data analysis: a tool was developed to assess HERV-K distribution across human populations [19]. Widely used tools for detecting HERV insertions in genomic data include MELT, STEAK, and RetroSeq. MELT and RetroSeq predict mobile element insertions by analyzing discordant read pairs and split reads [20,21], while STEAK utilizes HERV-K-specific partially aligned reads to identify both reference and non-reference loci [22].

Multiple computational tools are employed [23]. Common workflows involve RNA-seq read mapping to HERV-K reference loci using Salmon [24] or feature Counts [25], followed by differential expression analysis with DESeq2 [26]. To improve the resolution of ambiguously mapped reads, Telescope (version 1.0.3) applies a Bayesian framework to reassign reads to specific HERV-K loci [27]. Additionally, TEtranscripts (version 2.2.3) accounts for both uniquely mapping and multimapping reads, enabling quantitative retrotransposon expression estimation [28]. Integrated pipelines, such as GeneTEFlow (version 2020) (based on the SQuIRE library), combine read mapping, locus-level quantification, and differential analysis for comprehensive retrotransposon studies [29].

Verification of HERV-K presence may include quantitative PCR (qPCR) to validate RNA-seq findings [30] and cross-validation between WGS and RNA-seq results to exclude artifacts [31]. A systematic comparative analysis of retrotransposon detection tools, including Telescope (version 1.0.3) and TEtranscripts (version 2.2.3), highlighted the necessity of combining multiple methods to improve HERV-K detection accuracy due to inter-locus homology [32].

Estimation of locus-specific HERV expression from transcriptome data is complicated by the fact that sequences of different HERV groups differ slightly from each other. Quantitative real-time PCR is used to determine the level of HERV-K transcription in patients with CRC, as well as next-generation sequencing (NGS) to identify and characterize loci with differential expression of *gag*, *pol*, and *env* proteins [33]. Endoretroviral elements can also be detected using immunohistochemistry, qPCR and ELISA [34].

There are a number of studies devoted to the investigation of the relationship between HERV expression and cancer development, including CRC [35,36]. The increased expression of HERV in human colon carcinoma cells observed after exposure to cytostatic drugs in [34] indicates an existing relationship between chemotherapy resistance and HERV levels. A study [37] showed that CRC patients with HERV overexpression have a higher probability of tumor metastasis and chemotherapy resistance development.

A number of studies demonstrate the involvement of HERV in the immune response. In particular, deletion of ERV elements in HeLa cells led to disruption of the expression of IFN-inducible genes [38]. In epithelial ovarian cancer patients, HERV overexpression is associated with tumor infiltration by CD8 + PD1+ T cells [39]. It has also been shown that the level of HERV expression positively correlates with the level of expression of 72 genes involved in the response to double-stranded RNA in patients with CRC and other tumor types [40]. Analysis of the receptor spectrum of CD8 + T cells infiltrating colon adenocarcinoma in mice showed that the epitope of the endogenous retrovirus p15E is an immunodominant antigen [41]. These data suggest that tumors with HERV overexpression are characterized by increased immunogenicity.

Recent studies have deepened our understanding of the role of HERV-K in CRC. In a study [42], an association between hypomethylation of HERV LTR regions and increased *env* expression was demonstrated on a sample of 45 patients with CRC, which correlated with an aggressive tumor phenotype and poor prognosis (methylation analysis—bisulfite sequencing, expression—RT-qPCR). The in vitro and ex vivo models study (60 CRC samples) [43] revealed that oncogenic signaling pathways (RAS/MAPK) activate HERV-K transcription through epigenetic remodeling, promoting the formation of chimeric transcripts with cellular stress response genes (RNA-seq, ChIP-seq). These data complement the results of [33], where RNA-seq and RT-qPCR confirmed the role of HERV-K(HML-2) (*gag*, *pol*, *env*) as an immunogenic marker associated with the activation of interferon pathways in a sample of 274 patients with CRC. Modern studies emphasize the heterogeneity of HERV-K expression in CRC. The work [37] on a cohort of 287 patients identified a subgroup with HERV overexpression associated with low 5-year survival (HR = 2.1, *p* < 0.01). Locus-specific analysis [17] confirmed that activation of individual HERV-K(HML-2) proviruses can serve as a predictor of metastasis. These data are consistent with the results [40], where an increase in HERV-K(HML-2) transcripts correlated with the activation of IFN-γ and CXCL9/10 in the tumor microenvironment.

This study aims to evaluate the expression of HML-2 subgroup elements of the HERV-K family using RNA-seq data from paired tumor and normal intestinal tissues to identify retrotransposon activity patterns across molecular subtypes of colorectal cancer (CRC). Unlike previous studies based primarily on individual methodologies for mobile genetic elements expression estimation, our work proposes an integrative approach combining multiple bioinformatics tools (Telescope (version 1.0.3), TEtranscripts (version 2.2.3), GeneTEFlow (version 2020)) and data types (expression and methylation analysis). This strategy minimizes false-positive signals and identifies active HERV-K(HML-2) loci.

## 2. Results

### 2.1. RNA-Seq Results Processing

#### 2.1.1. Sample Classification by CMS Signatures

Transcriptomic and methylomic data from paired tumor and normal tissue samples were analyzed for 63 colorectal cancer (CRC) cases. Of these, 60 cases were successfully classified according to the Consensus Molecular Subtypes (CMSs). The clinical characteristics of patients included in the study, grouped by CMS, are summarized in Table 1.

As shown by the table, the CMS1 CRC cohort consists predominantly of samples with MSI-H status, which is consistent with the established features of the first consensus molecular subtype of CRC [5,37]. At the same time, MSI-H samples are also present among CMS3 and CMS4 [44]. Most CMS2–4 CRC samples were MSS, in accordance with the original classification [5]. In addition, CMS1 samples were predominantly right-sided tumors (4 out of 7) of stages I–III, whereas CMS2 and CMS4 mainly included patients with stage II disease, and CMS3 was primarily represented by stage III cases.

**Figure 1:**
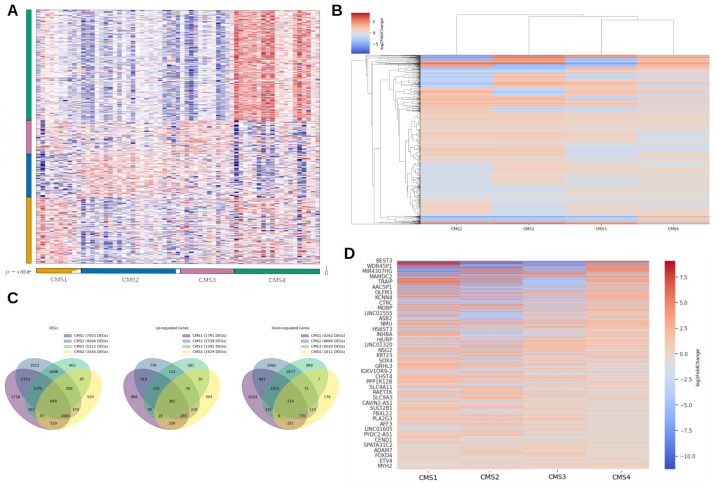
(**A**) Heatmap showing relative expression levels of CMS marker genes (vertical axis) with CMS classifications indicated below (horizontal axis); the white bar denotes statistical significance of prediction (*p*-values); (**B**) Heatmap showing the distribution of differential gene expression values. The heatmap presents genes with differential expression (|log_2_(FC)| ≥ 1, *p*-value < 0.5). A total of 13,366 genes is shown, with each stripe representing one value. Red lines indicate overexpression of the corresponding gene, and blue lines indicate underexpression. Each column corresponds to one of the four CMSs of CRC; (**C**) Venn diagram showing the intersection of DEGs among the four CMSs of CRC: intersection of all DEGs, intersection of DEGs with increased expression in each CMS, intersection of DEGs with decreased expression in each CMS; (**D**) Heatmap showing the distribution of differential gene expression values. The heatmap presents DEGs shared between CMSs (|log_2_(FC)| ≥ 1, *p*-value < 0.5), with each stripe representing one value. Red lines indicate overexpression of the corresponding gene, and blue lines indicate underexpression. Each column corresponds to one of the four CMSs of CRC.

The heatmap (Figure 1A) demonstrates clustering of CMS marker genes based on their expression levels, confirming structured differences between classification groups. The heatmap (Figure S1E) includes the results of mRNA gene set enrichment analysis.

#### 2.1.2. Differential Gene Expression by CMS

Differentially expressed genes (DEGs) were identified for each of the four CMS groups. As a result, a total of 7053 DEGs were found for CMS1 samples (padj < 0.05; |log_2_(FC)| ≥ 1), among which 2791 DEGs showed increased expression in tumor tissue compared to adjacent normal tissue, and 4262 DEGs showed decreased expression in tumors. A total of 9204 DEGs were identified in the group of CMS2 samples, including 2539 with increased expression in tumors and 6666 with decreased expression in tumors. CMS3 samples were characterized by 5312 DEGs, among which 1292 were upregulated and 4020 genes showed decreased expression levels. In CMS4 samples, a total of 3540 DEGs were identified, among which 1929 were upregulated and 1611 were downregulated.

Figure 1B shows the expression level distribution of genes that were differentially expressed in at least one of the CMSs in the form of a heatmap. A significant difference in gene expression is observed between CMSs: many genes are differentially expressed in only one subtype, i.e., DEGs are unique for a specific CMS.

For further analysis, the DEGs of each CMS were intersected with each other. The intersection results are shown in Figure 1C as a Venn diagram. Thus, 648 genes were found to be differentially expressed in all four CMSs. Figure 1D presents these 648 genes and their expression levels as a heatmap, showing that the direction of expression change differs between subtypes for these genes.

#### 2.1.3. Expression of HML-2 Loci

**Figure 2:**
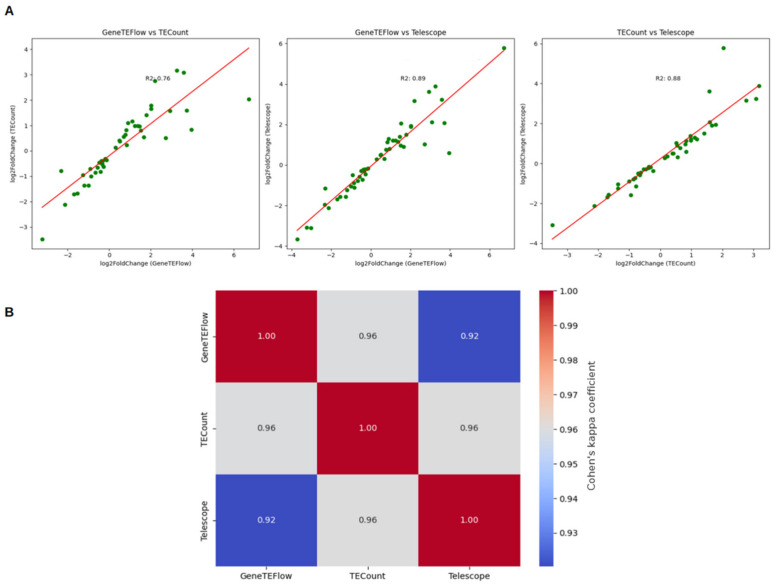
(**A**) Pearson correlation plots with R^2^ values. Each green point represents a locus and the red lines are superimposed to illustrate the convergence; (**B**) Heatmap of Cohen’s kappa coefficients for comparing the results of the three tools.

The results obtained using all three tools (GeneTEFlow (version 2020), TECount (version 2.2.3), Telescope (version 1.0.3)) for assessing HML-2 expression showed high convergence: the Pearson correlation coefficient for each tool exceeded 0.88 (Figure 2A).

Additionally, to further evaluate the consistency among the tools, Cohen’s kappa coefficient was calculated. This coefficient is used to assess the agreement between two classifiers, accounting for the probability of random chance. The following kappa values were obtained for all tool pairs (Figure 2B): GeneTEFlow (version 2020) vs. TECount (version 2.2.3): 0.96; GeneTEFlow (version 2020) vs. Telescope (version 1.0.3): 0.92; TECount (version 2.2.3) vs. Telescope (version 1.0.3): 0.96.

As a result of the analysis, lists of differentially expressed HML-2 loci were obtained. Using GeneTEFlow (version 2020) (HML-2_8p23.1b, HML-2_1q22, HML-2_22q11.23, HML-2_22q11.21, HML-2_9q34.11, and others), a total of 23 HML-2 loci with statistically significant differential expression changes (|log_2_(FC)| ≥ 1, padj < 0.05) were identified in the CMS1 colorectal cancer (CRC) sample group. Using Telescope (version 1.0.3) (HML-2_22q11.21, HML-2_8p23.1b, HML-2_8p23.1a, HML-2_1q22, HML-2_22q11.23, and others), 22 HERV-K loci with statistically significant differential expression changes (|log_2_(FC)| ≥ 1; padj < 0.05) were detected in the CMS1 CRC sample group. Using TECount (version 2.2.3), a total of 16 HML-2 loci with significant and sufficient expression changes in the CMS1 CRC (HML-2_8p23.1b, HML-2_1q22, HML-2_22q11.23, HML-2_22q11.21, HML-2_9q34.11, and others) were identified.

Further in this study, we consider HML-2 loci whose differential expression changes were statistically significant (padj < 0.05) according to all three tools, and for which the magnitude of expression changes met the threshold |log_2_FC| ≥ 1 according to at least two of the tools. Supplementary Figure S1A presents all 19 HML-2 loci identified in CMS1 CRC samples by all three tools.

Based on the results of the analysis using GeneTEFlow (version 2020), 19 statistically significant (padj < 0.05) differentially expressed loci (|log_2_FC| ≥ 1) (HML-2_11p15.4b, HML-2_12q14.1, HML-2_22q11.23, HML-2_22q11.21, HML-2_8p23.1b, and others) were identified in CMS2 CRC. Using TECount (version 2.2.3), a total of 16 significantly differentially expressed loci were identified (HML-2_11p15.4b, HML-2_22q11.23, HML-2_11q12.1, HML-2_8p23.1b, HML-2_10q24.2, and others). According to the results of Telescope (version 1.0.3), a total of 20 such loci were identified (HML-2_11p15.4b, HML-2_22q11.23, HML-2_4q32.1, HML-2_8p23.1b, HML-2_12q14.1, and others). Among these loci, 17 were found to be common across all three tools (Supplementary Figure S1B).

GeneTEFlow (version 2020) identified 15 statistically significant differentially expressed HML-2 loci (|log_2_FC| ≥ 1; padj < 0.05) (HML-2_22q11.21, HML-2_17p13.1, HML-2_22q11.23, HML-2_11q12.1, HML-2_8p22, and others) in samples of CMS3. A total of 14 statistically significant differentially expressed HML-2 loci (|log_2_FC| ≥ 1; padj < 0.05) were identified in CMS3 CRC samples Using TECount (version 2.2.3) (HML-2_22q11.23, HML-2_17p13.1, HML-2_11q12.1, HML-2_8p23.1b, HML-2_1q22, and others) and Telescope (version 1.0.3) (HML-2_22q11.21, HML-2_17p13.1, HML-2_22q11.23, HML-2_8p23.1b, HML-2_11q12.1, and others). Of these, only 12 loci were identified by all three tools (Supplementary Figure S1C).

GeneTEFlow (version 2020) identified a total of 7 statistically significant differentially expressed HML-2 loci (|log_2_FC| ≥ 1; padj < 0.05) (HML-2_22q11.23, HML-2_8p23.1b, HML-2_22q11.21, HML-2_11q12.1, HML-2_10p14, and others) in CMS4 samples. A total of 5 statistically significant differentially expressed (|log_2_FC| ≥ 1; padj < 0.05) HML-2 loci were identified Using TECount (version 2.2.3) (HML-2_8p23.1b, HML-2_21q21.1, HML-2_11q12.1, HML-2_10q24.2, HML-2_8q24.3a) and Telescope (version 1.0.3) (HML-2_4q32.1, HML-2_22q11.23, HML-2_11q12.1, HML-2_8p23.1a, HML-2_10p14). Among them, only two loci–HML-2_11q12.1 and HML-2_10p14–were identified by all three tools (Supplementary Figure S1D).

**Figure 3:**
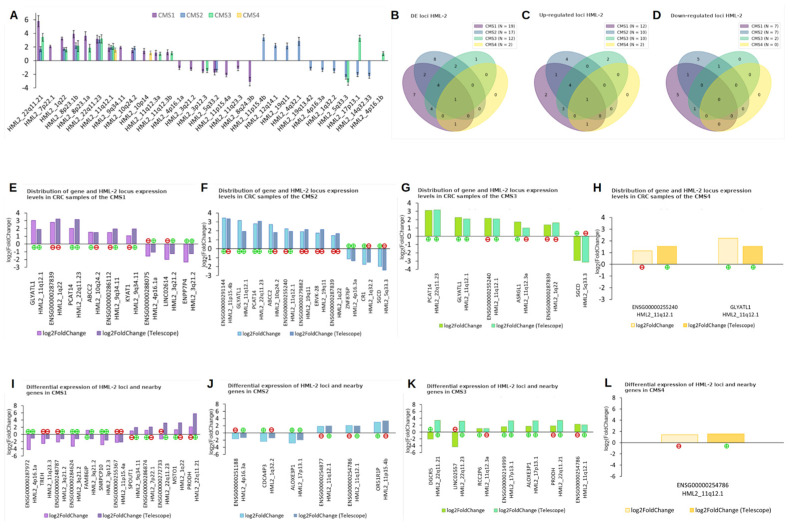
(**A**)—Bar plot showing the distribution of differential expression levels of HML-2 loci (padj < 0.05; |log_2_FC| ≥ 1) that were expressed in at least one of the four analyzed CMSs. Error bars on each column represent standard deviation values. Differential expression values of HML-2 loci were obtained using the Telescope tool; (**B**–**D**) Venn diagrams illustrating the overlap of differentially expressed loci among the four CMSs; (**C**) Overlap of loci with increased expression levels in CRC; (**D**) Overlap of loci with decreased expression levels relative to normal tissue; (**E**–**H**) Distribution of relative expression levels (padj < 0.05; |log_2_FC| ≥ 1) of genes and the HML-2 loci embedded within them. Differential expression values for HML-2 loci were obtained using the Telescope (version 1.0.3) tool. (**E**) Distribution of gene and HML-2 locus expression levels in CRC samples of CMS1; (**F**) Distribution of gene and HML-2 locus expression levels in CRC samples of CMS2; (**G**) Distribution of gene and HML-2 locus expression levels in CRC samples of CMS3; (**H**) Distribution of gene and HML-2 locus expression levels in CRC samples of CMS4. Green circles with a plus sign denote loci or genes located on the “+” (sense) DNA strand, while red circles with a minus sign indicate those located on the “–” (antisense) DNA strand; (**I**–**L**) Distribution of differential expression levels (padj < 0.05; |log_2_FC| ≥ 1) of HML-2 loci and adjacent genes. Differential expression values of HML-2 loci were obtained using the Telescope tool. (**I**) Differential expression of HML-2 loci and nearby genes in CMS1 colorectal cancer (CRC) samples; (**J**) Differential expression of HML-2 loci and nearby genes in CMS2 CRC samples; (**K**) Differential expression of HML-2 loci and nearby genes in CMS3 CRC samples; (**L**) Differential expression of HML-2 loci and nearby genes in CMS4 CRC samples.

Figure 3A presents a bar chart illustrating the relative expression levels distribution of all HML-2 loci that were expressed in at least one of the four analyzed CRC CMSs. 

As shown in the figure, many loci exhibit similar expression changes in CRC samples relative to normal tissues across multiple CMSs (HML-2_22q11.21, HML-2_1q22, HML-2_8p23.1b, and others), whereas some loci are uniquely expressed in only one subtype (HML-2_9q34.11, HML-2_11p15.4a, HML-2_11q23.3, and others). It is also worth noting the HML-2_17p13.1 locus, which shows contrasting expression patterns across CMSs compared to normal tissue: decreased expression in CMS2 and increased expression in CMS3.

These findings highlight differences among CMSs of CRC in the expression levels of specific HML-2 loci, underscoring their potential as candidate biomarkers.

The overlap analysis of differentially expressed HML-2 loci identified in CRC samples across the four CMSs (Figure 3B–D) enabled the identification of loci that were differentially expressed exclusively in CMS1 (increased expression observed for HML-2_7p22.1 and HML-2_9q34.11; decreased expression observed for HML-2_11p15.4a, HML-2_11q23.3, HML-2_8q24.3b, HML-2_4p16.1a, and HML-2_3q21.2). In CMS2, overexpression was detected for HML-2_11p15.4b, HML-2_12q14.1, HML-2_19q11, and HML-2_4q32.1, while underexpression was found for HML-2_19q13.42, HML-2_4p16.3a, HML-2_1q32.2, and HML-2_14q32.33. In CMS3, only HML-2_4p16.1b showed overexpression. No differentially expressed HML-2 loci were identified in CRC samples of CMS4. Based on the overlap across all CMSs, only one locus, HML-2_11q12.1, was found to be differentially expressed in all four subtypes.

#### 2.1.4. Identification of Genes Containing HML-2 Loci and Analysis of Their Expression

Taking into account data suggesting that the expression of an HML-2 locus may depend on the expression of the host gene [45], we performed an analysis to identify genes that harbor differentially expressed HML-2 loci. Analysis of CRC CMS1 samples revealed that differentially expressed HML-2 loci (identified using all three tools: GeneTEFlow (version 2020), Telescope (version 1.0.3), and TECount (version 2.2.3)) were located within 27 genes, including 8 protein-coding genes (such as *SSBP1*, *ASRGL1*, *ABCC2*, *KYAT1*, *GLYATL1*, and others), 15 long non-coding RNAs (lncRNAs) (e.g., *LINC02614*, *LINC00707*, *NEPRO-AS1*, *PCAT14*, etc.), and 4 pseudogenes (*ENPP7P10*, *ENPP7P4*, *ENPP7P15*, and one unnamed pseudogene). Among them, only 10 genes were differentially expressed (padj < 0.05; |log_2_FC| ≥ 1) in CMS1 CRC samples. In most cases, the gene and the embedded HML-2 locus were located on opposite DNA strands, although cases of co-strand localization were also observed. Figure 3E shows a bar plot illustrating the relative expression levels of the genes and the embedded HML-2 loci in CMS1 samples, with strand orientation (“+” or “–”) marked for both.

Analysis of CMS2 CRC samples showed that some differentially expressed HML-2 loci were located within 43 genes, including 15 protein-coding genes (*ZNF420*, *ZNF585A*, *CD48*, *ASRGL1*, *TTLL3*, etc.), 18 lncRNA genes (*LINC02614*, *FAM85B*, *ZNF876P*, *DHRS4L1*, *DHRS4-AS1*, and others), and 10 pseudogenes (*ENPP7P4*, *ENPP7P15*, *ENPP7P10*, *ENPP7P1*, *ENPP7P6*, etc.). Of these, 19 genes were found to be differentially expressed in CMS2. Figure 3F presents a bar plot showing the relative expression levels of the genes and the corresponding embedded HML-2 loci, with strand orientation annotations.

Analysis of CMS3 samples revealed that differentially expressed HML-2 loci in this group were located within 13 genes, including 5 protein-coding genes (*SGCD*, *ASRGL1*, *DEFB107B*, *GLYATL1*, and one unnamed gene), 7 lncRNA genes (*PCAT14*, *ENSG00000284294*, *ENSG00000287839*, *ENSG00000255240*, *ENSG00000263427*, and others), and one pseudogene (*ENSG00000246203*). Of these, only 6 genes were differentially expressed and are presented in a bar plot in Figure 3G, together with the expression levels of the embedded HML-2 loci.

Finally, analysis of CMS4 samples revealed that only one differentially expressed HML-2 locus—*HML-2_11q12.1*—was located within the protein-coding gene *GLYATL1* on the sense strand, and within the lncRNA *ENSG00000255240* on the antisense strand. Both genes, along with *HML-2_11q12.1*, were differentially expressed in CMS4 CRC samples. Figure 3H shows a bar plot illustrating the relative expression levels of these two genes and the embedded HML-2 locus.

Full data on DEG with HML-2 in CMSs 1–4 are presented in Supplementary Tables S1–S4.

#### 2.1.5. Identification of Genes near Differentially Expressed HML-2 Loci and Their Expression Analysis

Data suggests that changes in HML-2 expression levels may be associated with altered expression of genes located in proximity to HML-2 loci [46]. Therefore, we performed a search for genes located near differentially expressed HML-2 loci in each of the four CMSs.

In CMS1 samples, a total of 106 genes were located within 50,000 bp of differentially expressed HML-2 loci, of which only 13 genes were differentially expressed. Among these 13 genes, four were protein-coding (*TREH*, *SPOUT1*, *MSTO1*, and *PRODH*), seven were long non-coding RNAs (*FAM86JP*, *ENSG00000287972*, *ENSG00000248787*, *ENSG00000284624*, *ENSG00000255367*, and others), and one was a pseudogene (*SNRPCP10*). A bar chart showing the distribution of relative expression values of the HERV-K loci and the genes located near them is presented in Figure 3I.

Analysis of CMS2 samples showed that 66 genes were located near HML-2 loci that were differentially expressed in this subtype. A total of 20 of these genes were also differentially expressed. Among these 20 genes, nine were protein-coding (*ALOX12B*, *LARP1*, *MSTO1*, *OR52K2*, *OR52K1*, and others), two were immunoglobulin genes (*IGHG2* and *IGHG4*), three were long non-coding RNAs (*DGCR5*, *ENSG00000280418*, and *LINC02557*), and six were pseudogenes (*CDCA4P3*, *ALOXE3P1*, *OR51R1P*, and others). A bar chart with the relative expression levels of the described HML-2 loci and associated genes is shown in Figure 3J.

In CMS3 samples, 57 genes were found in the vicinity of differentially expressed HML-2 loci, seven of which also exhibited differential expression. Among these seven genes, only one, *PRODH*, was protein-coding. This group also included three long non-coding RNAs (*DGCR5*, *LINC02557*, and *ENSG00000214999*) and three pseudogenes (*RCC2P6*, *ALOXE3P1*, and *ENSG00000254786*). The relative expression levels of these loci and nearby genes are shown in Figure 3K.

Analysis of CMS4 colorectal cancer samples revealed only seven genes located near differentially expressed HML-2 loci, of which only one pseudogene, *ENSG00000254786*, was differentially expressed. It is located near the *HML-2_11q12.1* locus but on the opposite strand. Their relative expression levels are presented in Figure 3L.

Consider DEGs located near HERV-K and detected in several CMSs (full data are presented in Supplementary Tables S5–S8). The *MSTO1* gene is overexpressed in samples of CMS1 and CMS2. The gene is located on the sense strand, while HML-2_1q22, which is overexpressed in both subtypes, is on the antisense strand. The protein encoded by this gene is localized in mitochondria and is involved in their morphogenesis [47]. Overexpression of HML-2_1q22 (ERVK-7) positively correlates with macrophage activation and type I interferon response activation [48]. Overexpression of ERVK-7 has also been detected in lung adenocarcinoma tissues [49].

The *FAM86JP* gene is overexpressed in samples of CMS1 and CMS2. The gene is located on the sense strand together with HML-2_3q21.2, which is hypoexpressed in CMS samples. This is a pseudogene with unknown function. The *PRODH* gene is overexpressed in samples of CMS1 and CMS3. The gene is on the antisense strand, while HML-2_22q11.21, which is also overexpressed in CMS1 and CMS3 samples, is on the sense strand. This gene encodes proline dehydrogenase 1, which can both suppress and promote tumor growth under certain conditions. Proline dehydrogenase catalyzes the formation of reactive oxygen species, leading to apoptosis of CRC cells [50]. Under stress, proline derived from collagen degradation is further metabolized by proline dehydrogenase to glutamate and α-ketoglutarate, which participate in the formation of ATP necessary for CRC cell survival [51]. Proline derived from collagen also promotes the survival and proliferation of pancreatic adenocarcinoma cells under nutrient deprivation, as shown by Olivares et al. [52]. The *DGCR5* gene is hypoexpressed in CMS2 and CMS3 samples. The gene is located on the sense strand together with HML-2_22q11.21, which is overexpressed in both subtypes. This gene encodes a lncRNA whose expression is reduced in CRC tissues. Overexpression of *DGCR5* in vitro led to inhibition of CRC cell proliferation through interaction with miR-21 and reduction in its expression [53]. Increased expression of HML-2_22q11.21 (ERVK-24), located between *PRODH* and *DGCR5*, was detected in germ cell tumor cells [46]. The *ALOXE3P1* gene is hypoexpressed in CMS2 samples and overexpressed in CMS3 samples. The gene is located on the sense strand together with HML-2_17p13.1, which is hypoexpressed in CMS2 and overexpressed in CMS3. This gene is a pseudogene with unknown function. The *LINC02557* gene is also hypoexpressed in CMS2 and CMS3 samples. This gene is on the antisense strand, while HML-2_22q11.23, which is upregulated in both subtypes, is on the sense strand. This gene encodes a lncRNA whose functions are currently uncharacterized.

DEGs located near HERV-K and detected only in CMS1 include *TREH*, *ENPP7P11*, *SPOUT1*, and *SNRPCP10*. The *TREH* gene and HML-2_11q23.3 are hypoexpressed and located on the antisense strand. Polymorphisms in the *TREH* gene are associated with the risk of glioma and glioblastoma [54,55]. The ENPP7P11 pseudogene is hypoexpressed and located on the sense strand together with HML-2_4p16.1b, which is not a DEG. The function of ENPP7P11 is unknown. The *SPOUT1* gene on the antisense strand and HML-2_9q34.11 on the sense strand are overexpressed. This gene encodes a kinetochore-associated protein [56]. The *SNRPCP10* pseudogene is hypoexpressed and located on the sense strand together with hypoexpressed HML-2_3p12.3. The function of this pseudogene is unknown.

DEGs located near HERV-K and detected in CMS2 samples include *LARP1*, *CIDEC*, *OR52K1*, *CD200R1*, *OR51R1P*, *CARMIL3*, *LIPH*, *FAM66D*, *ALOX15B*, *ALOX12B*, *ZNF732*, *WEE2*, and *IGHG2*. The *LARP1* gene is overexpressed and located on the sense strand, while hypoexpressed HML-2_5q33.2 is on the antisense strand. Ye et al. showed that *LARP1* overexpression is associated with poor prognosis in CRC [57]. The *CIDEC* gene is hypoexpressed and located on the antisense strand together with HML-2_3p25.3, which is not a DEG. The protein encoded by this gene is specifically expressed in adipose tissue and is associated with adipocyte metabolism [58]. The *OR52K1* and *OR51R1P* genes are overexpressed. They are located on the sense strand, while their shared overexpressed HML-2_11p15.4b is on the antisense strand. *OR51R1P* is a pseudogene with unknown function, and OR52K1 encodes an olfactory receptor-associated protein [59]. The *CD200R1* gene is hypoexpressed and located on the antisense strand together with HML-2_3q13.2, which is not a DEG. Reduced expression of this gene is associated with increased overall survival in rectal cancer patients compared to those with elevated expression [60]. The *CARMIL3* gene is overexpressed and located on the sense strand, while HML-2_14q11.2, not a DEG, is on the antisense strand. The product of this gene may promote degradation of the tumor suppressor p53, thereby stimulating HCC cell proliferation [61]. The *LIPH* gene is hypoexpressed and located on the antisense strand together with HML-2_3q27.2, which is not a DEG. Overexpression of this gene was detected in pancreatic tumor tissue and was associated with poor prognosis [62]. The *FAM66D* gene is hypoexpressed and located on the sense strand, while HML-2_8p23.1d, which is not a DEG, is on the antisense strand. The function of *FAM66D* is unknown. The *ALOX15B* and *ALOX12B* genes are hypoexpressed. *ALOX15B* is on the sense strand, and *ALOX12B* is on the antisense strand. HML-2_17p13.1, which is also hypoexpressed, is on the sense strand for both genes. Some mutations in *ALOX15* are associated with increased CRC risk [63]. Increased *ALOX12B* expression was found in patients with metastatic gastric cancer and was associated with poor prognosis [64]. The *ZNF732* gene is hypoexpressed and located on the antisense strand, while HML-2_4p16.3a, which is also hypoexpressed, is on the sense strand. Reduced *ZNF732* expression has been found in laryngeal carcinoma tissues [65]. The *WEE2* gene is hypoexpressed and located on the sense strand, while HML-2_7q34, not a DEG, is on the antisense strand. This gene encodes a kinase that is a key regulator of meiosis [66]. The *IGHG2* gene is overexpressed and located on the antisense strand, while HML-2_14q32.33, which is hypoexpressed, is on the sense strand. Increased expression of this gene has been observed in tumor samples from CRC patients [67].

The following DEGs were found in CMS3 samples: *CSPG4P11*, *DNM1P41*, *RCC2P6*, *EFL1P1*, and *CR1L*. The *CSPG4P11* pseudogene is hypoexpressed and located on the sense strand together with hypoexpressed HML-2_15q25.2. The function of this pseudogene is unknown. The *DNM1P41* gene is hypoexpressed and located on the antisense strand, while HML-2_15q25.2, which is also hypoexpressed, is on the sense strand. Researchers suggest that increased expression of this lncRNA may be used as a marker for HCC development in the context of HBV infection, but its functions are uncharacterized [68]. The *RCC2P6* pseudogene is overexpressed and located on the sense strand; its function is unknown. HML-2_11q12.3a, which is also overexpressed, is on the antisense strand. The *EFL1P1* pseudogene is hypoexpressed and located on the sense strand together with hypoexpressed HML-2_15q25.2. The function of this pseudogene is unknown. The *CR1L* gene is hypoexpressed and located on the sense strand, while hypoexpressed HML-2_1q32.2 is on the antisense strand. This gene is actively expressed in hematopoietic and fetal lymphoid tissues and is presumed to play a role in lympho- or hematopoiesis [69]. This gene is also associated with schizophrenia [70]. It is also a paralog of the CR1 gene, which encodes a complement system receptor involved in innate and adaptive immune responses [71].

Only one DEG was found in the CMS4 –*MTHFD1L*. This gene is overexpressed and located on the sense strand together with HML-2_6q25.1, which is not a DEG. Increased expression of *MTHFD1L* was detected in CRC tissues, and suppression of this gene’s expression via RNA interference in CRC cell lines led to inhibition of proliferation, migration, and invasive activity of tumor cells [72].

### 2.2. Association Between DNA Methylation Levels and Gene Expression Within HML-2 Containing Genes

As a result of methylation analysis across different CMSs, overlapping genes and special aspects of their expression were identified and integrated to provide a comprehensive overview of methylation in these subtypes.

The total number of identified methylation sites in CMS samples was as follows: in CMS1, 242 methylation sites were detected across 17 genes; in CMS2—367 sites in 19 genes; in CMS3—145 sites in 10 genes; and in CMS4—48 sites in 7 genes (Supplementary Tables S9–S12). These subtypes revealed genes with both upregulated and downregulated differential expression, with some genes appearing in multiple subtypes.

Genes with downregulated expression across different CMSs include: *CD48*, *CR1*, *FAM85B*, *FGL1*, *LHFPL3*, *MEI4*, *SGCD*, *TRPC6*, *ZNF876P*—shared among CMS1, CMS2, and CMS3; *DHRS4L1*, *ENPP7P4*, *LHFPL3-AS1*, *LINC02614*—found only in CMS1; *LINC00707*, *ZNF600*—specific to CMS2; *NEPRO-AS1*, *ZNF876P*—found only in CMS3.

Genes with upregulated expression include: *ABCC2*, *GLYATL1*—common to CMS1, CMS2, and CMS3, *KYAT1*—found only in CMS1, *LHFPL3-AS1*—observed only in CMS2; *MIR548XHG*—specific to CMS4.

For each subtype, methylation sites located in gene promoter regions were also identified.

In CMS1, 37 promoter-associated methylation sites were identified within the genes *ABCC2*, *CD48*, *CR1*, *ENPP7P4*, *FAM85B*, *FGL1*, *GLYATL1*, *KYAT1*, *LHFPL3*, *SSBP1*, *TTLL3*, and *ZNF420*. Among these, 11 sites exhibited hypermethylation in the promoters of *TTLL3*, *LHFPL3*, *CR1*, and *FAM85B*, while 6 sites were hypomethylated in the promoters of *CD48*, *ABCC2*, and *GLYATL1* (Figure S2A, Supplementary Materials).

In CMS2, 35 methylation sites were found in promoter regions of *ABCC2*, *CR1*, *FAM85B*, *FGL1*, *GLYATL1*, *LHFPL3*, *MEI4*, *SSBP1*, and *TRPC6*, of which 16 sites were hypomethylated in the promoters of *ABCC2*, *FGL1*, *TRPC6*, and *FAM85B*, and 10 sites were hypermethylated in the promoters of *TRPC6*, *CR1*, *LHFPL3*, and *MEI4* (Figure S2B, Supplementary Materials).

In CMS3, 15 methylation sites were located in promoter regions of *TRPC6*, *CR1*, *SGCD*, *MEI4*, *NEPRO-AS1*, and *GLYATL1*, among which 11 sites were hypermethylated in the promoters of *TRPC6*, *CR1*, *SGCD*, *MEI4*, *NEPRO-AS1*, and *GLYATL1*, and 2 sites were hypomethylated in the promoter region of *TRPC6* (Figure S2C, Supplementary Materials).

In CMS4, 7 methylation sites were found in promoter regions of *ABCC2*, *GLYATL1*, *SSBP1*, *FGL1*, and *ENPP7P4*, of which one site was hypomethylated in the promoter region of a long non-coding RNA, and no hypermethylated sites were identified (Figure S2D).

**Figure 4:**
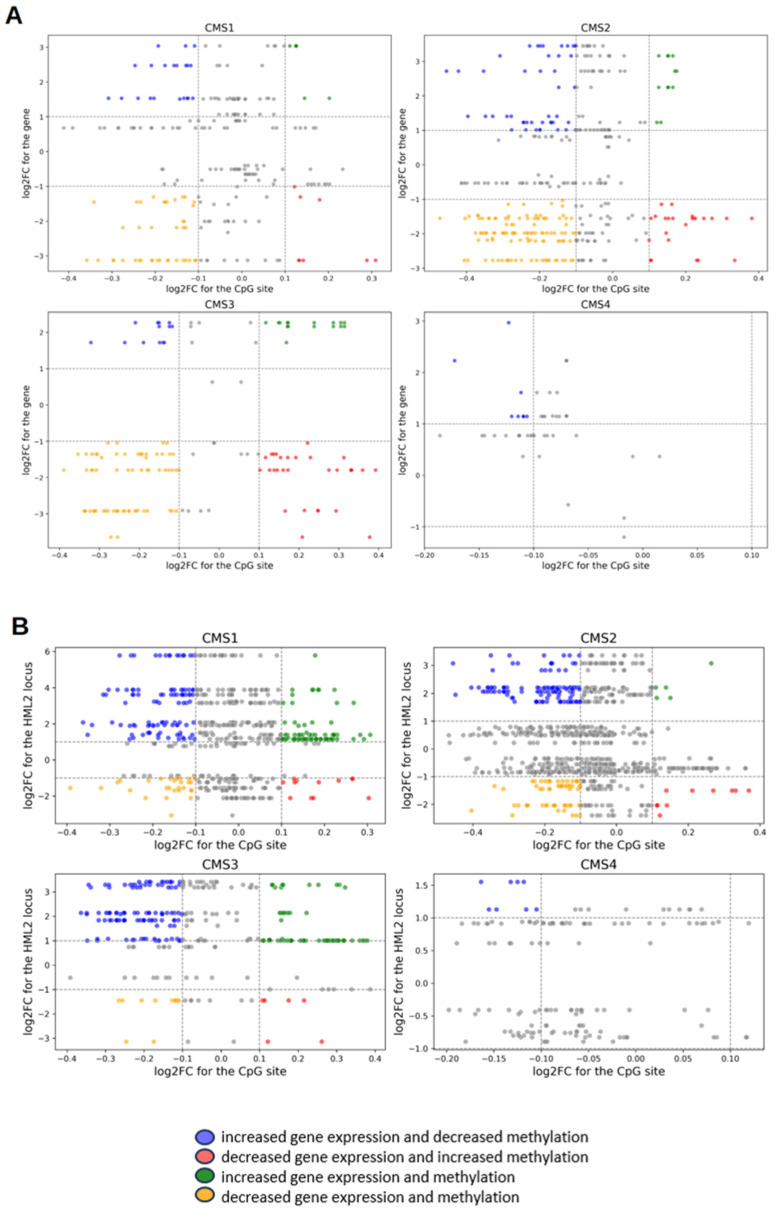
(**A**) Scatter plot of average DNA methylation difference versus gene expression change (log_2_FC); (**B**) Scatter plot of average DNA methylation difference versus HML-2 locus expression change (log_2_FC). Each point represents a CpG–gene pair. Plots are organized according to CMS. Blue dots correspond to regions with increased gene expression and decreased methylation. Red dots indicate regions with decreased gene expression and increased methylation. Green dots represent regions with both increased methylation and gene expression. Orange dots denote regions with both methylation and gene expression decreased. Grey dots correspond to regions with no differential expression and no differential methylation.

Figure 4A presents a volcano plot displaying the distribution of a scatterplot of the average difference in methylation versus gene expression changes (log_2_FC). Each point on the graph represents a CpG-gene pair, with gene expression changes plotted on the *Y*-axis and methylation changes on the *X*-axis. The analysis shows that, in each CMS, a substantial number of points fall within the region characterized by gene downregulation and increased methylation.

In addition, methylation sites located within 10,000 bp of HML-2 loci were analyzed separately. Figure 4B presents a plot illustrating a scatter diagram of the average DNA methylation difference versus changes in HML-2 locus expression (log_2_FC). Each point on the graph represents a CpG–HML-2 pair. The analysis revealed that most points are concentrated in the region characterized by hypomethylation and increased expression of HML-2 loci.

In total, 569 methylation sites were found near HML-2 loci in CMS1, 923 in CMS2, 286 in CMS3, and 138 in CMS4.

The analysis showed that the number of DEGs, differentially methylated genes, as well as differentially expressed HML-2 elements, gradually decreases from CMS1 to CMS3. This corresponds to previously established data on more pronounced immune activity in the CMS1 and a more “cold” immunophenotype in CMS3.

HML-2 elements with increased methylation are more often localized within DEGs with also increased expression, which possibly may indicate co-regulation of retroviral loci and their genomic context. For example, HML-2_9q34.11 is part of the *KYAT1* gene in CMS1, HML-2_10q24.2 is in the *ABCC2* gene in CMS1 and CMS2, and HML-2_11q12.1 is in *GLYATL1*, which is hyperexpressed in all four subtypes. At the same time, hypoexpressed HML-2 elements are often located inside downregulated DEGs such as *ENPP7P4*, *SGCD*, *LINC02614*, *CR1*, and *LHFPL3*.

A correlation between expression and methylation levels is also observed: genes with increased methylation generally demonstrate reduced expression. Examples include *CR1*, *LHFPL3*, *MEI4*, *TRPC6*, and *SGCD*, which have increased methylation in different CMSs and simultaneously show decreased transcription levels. The opposite situation (decreased methylation and increased expression) is less common but also observed, as in the cases of *ABCC2* and *FGL1*.

Among all DEGs containing HML-2 elements, only five genes are directly associated with colorectal cancer (CRC): *TTLL3*, *ABCC2*, *ZNF420*, *CD48*, and *FGL1*. Their involvement in CRC pathogenesis is supported by several independent studies. *CD48* is an immune response molecule, hypoexpressed in CMS1 and CMS2. It is associated with microsatellite instability, tumor mutational burden, and patient survival in CRC [73,74,75,76]. *FGL1* is a ligand of the inhibitory receptor *LAG3*, hypoexpressed in CMS1 and CMS2 and hyperexpressed in CMS4. It may suppress antitumor immunity through T cell exhaustion [77,78,79,80]. *TTLL3* is an enzyme regulating microtubule stability, whose expression is decreased in all subtypes. Its absence may increase epithelial cell proliferation [81]. *ABCC2* is a transporter involved in detoxification. It is hyperexpressed in CMS1, CMS2, and CMS4, consistent with data on its increased expression in CRC from other studies [82,83]. *ZNF420* (*APAC*) is a factor negatively regulating p53. Its reduced expression in CMSs 1–3 may be associated with a favorable prognosis in CRC [84].

Additional interest is represented by *KYAT1* and *CR1*. *KYAT1* participates in kynurenine metabolism, influencing the immune response through T cell suppression, activation of the PI3K–Akt cascade, and induction of immunosuppression. *CR1* is a complement system component, hypoexpressed in CMSs 1–3, whereas in other studies its hyperexpression is noted in other cancer types [85,86,87].

### 2.3. Relationship Between Differential Methylation of CpG Sites and Differential Expression of Genes Containing the HML-2 Locus and Located Within 3000 bp of the CG Site

After performing the filtering (as described in Methods), the data set for the CMS1 subtype includes 35 CpG site-gene pairs, which include 35 unique CpG sites and 8 unique genes. The CMS2 dataset contains 170 CpG site-gene pairs, including 158 unique CpG sites and 17 unique genes. For the CMS3 subtype, 123 pairs were filtered, including 115 unique CpG sites and 9 unique genes. There were 6 pairs remaining in the CMS4 data, including 6 unique sites and 2 unique genes.

The results of correlation tests between differential methylation of CpG sites and differential gene expression are shown in Table 2. A statistically significant correlation is found for CMS2 and CMS3. Correlation coefficient Rho is positive in both cases, indicating an increase in differential gene expression with increased differential methylation of CpG sites.

The regression model described in Section 4.12 was applied to the data for CMSs 1–3. The model is not applicable to the CMS4 data because all gene-site CpG pairs are located on the same chain and all CpG sites are not located in the promoter in these data. A statistically significant (*p*-value = 0.03) linear relationship between differential methylation of CpG sites and differential gene expression was found only for CMS3. The corresponding regression coefficient is positive, which is consistent with the results of the correlation test. In addition, for CMS3, a statistically significant effect of CpG site location on the relationship between differential methylation and differential expression was found (Table S13, Supplementary Materials). In particular, the location of the differentially methylated CpG site in the gene promoter changes the linear relationship between differential methylation and differential expression from positive to negative (*p*-value < 0.1).

### 2.4. Relationship Between Differential Methylation of CpG Sites and Differential Expression of HML-2 Loci Located Within 10,000 bp of Each Other

After the filtering described in Section 4.11, the data set for CMS1 includes 215 CpG site-HML-2 locus pairs, which include 203 unique CpG sites and 19 unique HML-2 loci. The CMS2 dataset contains 179 CpG site-HML-2 locus pairs, including 176 unique CpG sites and 17 unique HML-2 loci. For CMS3, 193 pairs were filtered, including 178 unique CpG sites and 12 unique loci. There were 8 pairs remaining in the CMS4 data, including 8 unique sites and 2 unique loci.

For each of the CMSs, an inverse relationship was found between differential methylation of CpG sites and differential expression of HML-2 loci. For all subtypes except CMS4, the relationship was statistically significant. The values of the correlation coefficient Rho are presented in Table 3.

Regression analysis showed that the relationship between differential methylation of CpG sites and differential expression of HML-2 loci was not affected by the location of the locus and site on the same or opposite chains. The regression coefficient, associated with $X_{1i}$, is negative for each of the four subtypes, but it is statistically significant only for CMS2. This indicates that there is a linear negative relationship between differential methylation of CpG sites and differential expression of HML-2 loci. The full results of the regression analysis, including robustness values of the models, are presented in the Tables S13 and S14, Supplementary Materials.

### 2.5. Quantitative Assessment of Tumor Immune Microenvironment

We applied the xCell algorithm to assess gene expression of special signatures indicating possible tumor infiltration by specific types of immune and stromal cells. This allowed us to identify different patterns of cell population distribution in molecular subtypes (Figure S2A).

CMS1 exhibits increased infiltration by lymphocytes associated with both innate (macrophages, NK cells) and adaptive immunity (B cells, CD8 and CD4 memory T cells, T helpers, and T regulatory cells). In addition, a notable increase in γδ T cells, which play a role in both forms of immunity, is observed in this subtype [88]. This apparently relates to the microsatellite instability which is characteristic of this subtype and leads to an increased number of neoantigens, triggering an active immune response [89]. The various B cell populations observed in this subtype may exhibit both pro- and anti-tumor activity [90]. Furthermore, M1 macrophages can suppress tumor cell growth, whereas M2 macrophages, conversely, promote tumor proliferation [91]. Other lymphocytes, except for T regulatory cells capable of suppressing anti-tumor immunity, possess anti-tumor properties [92,93,94].

In contrast, tumors of CMS4 are mostly infiltrated by cell populations characterized by pro-tumor activity, namely M2 macrophages, neutrophils, and T regulatory cells. This subtype shows an increased content of endothelial cells, smooth muscle cells, and fibroblasts necessary for angiogenesis and contributing to the poor prognosis typical of this subtype [95]. However, a certain number of leukocytes with anti-tumor properties, namely CD4 and CD8 T cells, are also observed in this subtype [9,96].

CMS2 tumors are characterized by reduced immune cell infiltration and apparently have low immunogenicity, which is consistent with previous studies [95]. A relatively increased number of T helpers was found in this subtype.

CMS3 tumors are also considered to have low immunogenicity [95]. We observe a trend toward increased content of B cells and NKT cells.

For all subtypes, an increased content of various groups of dendritic cells was registered. Dendritic cells generally exhibit anti-tumor activity, but under certain conditions their function can be impaired, even manifesting immunosuppressive pro-tumor activity [97,98]. Populations of epithelial cells are also present in all CMSs, which is typical of carcinomas [99].

For intergroup comparison and identification of statistically significant differences between subtypes (differences of at least one subtype from the others) the Kruskal–Wallis test was applied (Table S15, Supplementary Materials). Then the results were evaluated using the nonparametric Dunn’s post hoc test, which by default performs all pairwise comparisons using ranks from the Kruskal–Wallis test (Table S16, Supplementary Materials). The Benjamini–Yekutieli correction was used to adjust the *p*-values. Thus, 8 cell populations with adjusted *p*-value < 0.05 were identified. These are different types of dendritic cells (DC, aDC, cDC), with an increased number of dendritic cells overall (DC) observed in CMS1 and CMS4 (padj = 0.0247), as well as activated dendritic cells (aDC) (padj = 0.0247). Increased content of conventional dendritic cells (cDC) was found in CMS3 and CMS4 (padj = 0.0311). Increased numbers of macrophages (padj = 0.0146) and M1 macrophages (padj = 0.0294) were also recorded in CMS1 and CMS4. The content of fibroblasts (padj = 0.004) and endothelial cells (padj = 0.0489), as expected, is increased in CMS4. The monocyte population is most represented in CMS4 (padj = 0.0025).

### 2.6. HML-2 Expression Assessed by RT-PCR

To assess the expression levels of two *HERV-K* genes—*gag* and *env*—in tumor and normal colorectal tissues, quantitative PCR (qPCR) was performed. The qPCR data are considered as a complementary method allowing the evaluation of overall *HERV-K* protein expression.

**Figure 5:**
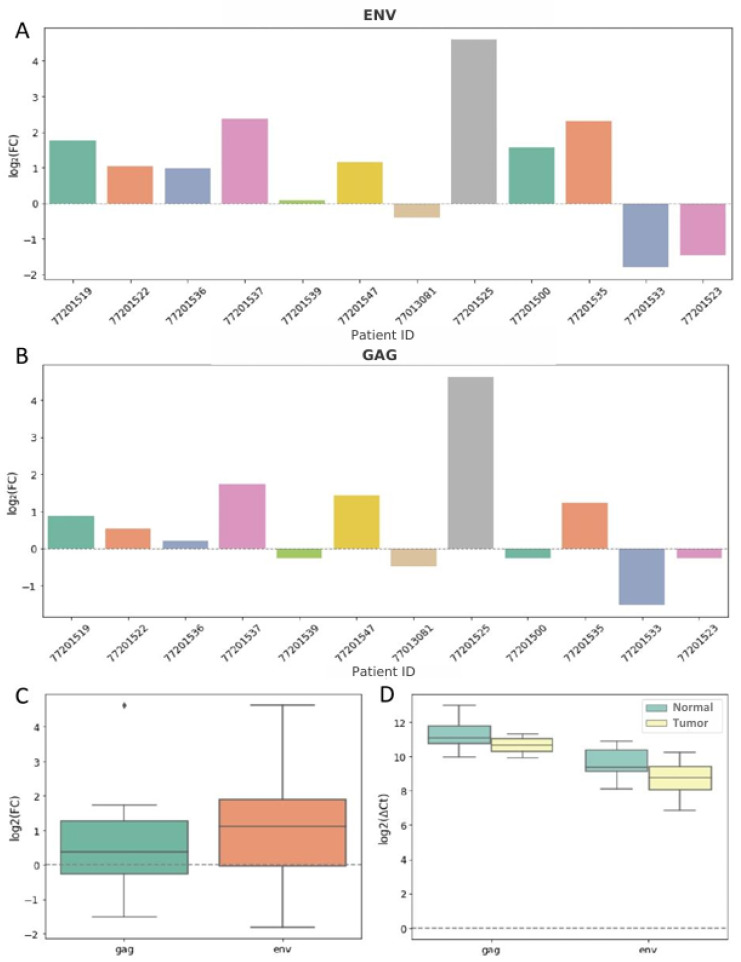
(**A**,**B**) Bar charts of log_2_(fold change) expression of env and gag in tumor tissue compared to paired normal tissue for each colorectal cancer patient. The log_2_(FC) values were calculated as log_2_(2^−ΔΔCt^). Positive values correspond to increased expression in tumor tissue, while negative values indicate decreased expression; (**C**) Boxplot of log_2_(FC), where FC (fold change) = 2^−ΔΔCt^, for the gag and env genes in tumor samples. Positive log_2_(FC) values indicate elevated expression in tumors compared to normal tissue; (**D**) Boxplot of log_2_(ΔCt) for the gag and env genes in normal (green) and tumor (yellow) tissues of colorectal cancer patients. The ΔCt parameter was calculated as the difference between the Ct values of the target and reference genes. Since ΔCt is inversely proportional to expression level, lower values correspond to higher expression.

In most cases, *env* expression was higher in tumor tissue compared to normal tissue (Figure 5A). The mean ΔCt (tumor − normal) for *env* was −0.92 ± 1.16, indicating elevated expression in tumors. The average relative change in expression (2^−ΔΔCt^) in tumors was 2.12-fold, with a median of 1.97 (Figure 5C,D).

In most paired samples, a trend toward increased *gag* expression in tumor tissue was observed (Figure 5B). Mean ΔCt (tumor − normal) was (−0.52) ± 1.30 (M ± SD), indicating increased *gag* expression in tumor tissue compared to normal tissue. The average relative change in expression (2^−ΔΔCt^) in tumors compared to normal tissue was 1.63-fold, with a median of 1.15 (Figure 5C,D).

## 3. Discussion

In the present study we performed a comprehensive analysis of HERV-K(HML-2) loci and genes expression in CRC samples using current bioinformatics tools and RT-PCR methods in combination with DNA methylation analysis. To evaluate HML-2 expression in CRC, we employed three computational tools: the TECount module of TEtranscripts (version 2.2.3) [28], Telescope (version 1.0.3) [27], and GeneTEFlow (version 2020) [29]. The results demonstrated high concordance between the tools: Pearson’s correlation coefficient for HML-2 expression reached ~0.88. Similar consistency has been reported in other studies. For instance, Liu et al. demonstrated a correlation of ~0.97 between GeneTEFlow and SQuIRE for gene expression analysis [29]. Both tools enable comprehensive evaluation by simultaneously assessing gene and retrotransposon expression, as confirmed by their integration into a unified pipeline for studying tissue-specific regulation of mobile elements [29]. Likewise, Bendal et al. noted that Telescope provides more accurate resolution of ambiguous reads compared to TEtranscripts, although both methods detect similar transposon expression patterns [27].

The use of multiple tools for retrotransposon expression analysis is becoming common practice in contemporary research. For example, Goerner-Potvin et al. employed TEtranscripts alongside SalmonTE, which revealed comparable differential expression patterns with a correlation of 0.97 [100]. In our study, the intersection of data from TEtranscripts (version 2.2.3), Telescope (version 1.0.3), and GeneTEFlow (version 2020) not only confirmed the reliability of identified HML-2 loci but also minimized algorithm-specific artifacts. Our research focused on comparative analysis of HML-2 expression as an activation marker of known reference retrotransposons, which aligns with the current capabilities of the employed bioinformatics tools. Further study will allow us to expand the analysis by incorporating de novo insertion detection methods and integrating DNA and RNA sequencing data to enhance accuracy and comprehensiveness of the study.

HML-2 expression assessment by RT-PCR demonstrated elevated expression of HML-2 *gag* and *env* genes in CRC samples compared to normal adjacent tissues, consistent with findings by Kang et al. (2023) [33], who also reported increased HERV-K protein levels in tumors. However, unlike our paired-samples study analyzing matched patient samples, Kang et al. employed a cohort-based approach with a larger sample size, which may account for some discrepancies in statistical significance. For accurate comparison with bioinformatics-derived data, locus-specific HERV expression analysis is required, involving primers selected for the certain retrotransposon loci. Future studies should employ expanded cohorts to enhance reliability and reproducibility of the results.

Published data support HERV involvement in carcinogenesis through multiple mechanisms. In colon adenocarcinomas, over 400 differentially expressed HERV loci overlap with protein-coding or long non-coding RNAs [17]. More than 100 of these loci coincide with or flank DEGs, suggesting potential co-regulation. HERV-K *gag* and *pol* proteins exhibit differential expression in CRC patients’ blood and correlate with genes associated with proliferation and survival (e.g., *LHFPL3*, *KYAT1*, *CR1*) [33]. A statistically significant association exists between specific HERV family expression and tumor localization (left/right-sided) [42]. LTR10 repeat elements within HERVs may function as MAPK/AP1 cascade-activated enhancers, driving pathological transcriptional reprogramming [43].

Immune pathway analysis (GSEA/ClueGO) highlights key roles for: TLR, IL-17, NETosis, and RIG-I-like pathways—all linked to innate immunity and may indicate the viral mimicry induced by HERVs.

However, it should be taken into account that observed pathway alterations primarily involve effector molecules (*CXCL1*, *CXCL8*, *IL6*, *CCL2*, *CSF2*, *CSF3*) rather than upstream receptors, suggesting immune cell infiltration rather than intrinsic tumor activity.

Potential roles of identified DEGs located near HERV-K can be considered in pathogenesis of CRC and other types of cancer. Overexpression of HML-2_1q22 was detected in CMS1 and CMS2 samples in our study. The analysis by Russ et al. demonstrated that HML-2_1q22 (*ERVK-7*) is activated by IFN-γ and subsequently activates the type I interferon response [48]. The type I interferon response may be involved in mediating both antitumor and protumor immune processes in cancer tissues [101]. This may also indicate viral mimicry, which is accompanied by activation of the interferon response [85], and it may be especially characteristic of CMS1, which is associated with the activation of the antitumor immune response [5]. Similarly, our results also indicate overexpression of HML-2_22q11.21 (*ERVK-24*) in CMS1–3 samples. Increased expression of *ERVK-24* has also been observed in germ cell tumor cell lines, suggesting its potential role in carcinogenesis [46]. The uneven representation of the subtypes in the cohort is a limitation of this study. It is a subject to correct in further work by adding additional clinical cases to the study.

Expression analysis of HERV-K located near DEGs in CRC samples of various CMSs revealed that HERV-K elements with both hypo- and hyperexpression are found in each CMS in approximately equal proportions. At the same time, there is a certain tendency towards a decrease in the number of such elements from CMS1 to CMS3 for HERV-K located within DEGs, as shown in section “Comparison of DNA methylation levels and expression of genes containing HML-2”. It may indicate differences in the mechanisms of HERV-K regulation depending on their localization within the genes or near the genes. The obtained data did not reveal clear trends in the association between the expression of HERV-K located near DEGs and specific molecular subtypes of CRC.

Analysis of the biological functions of the identified DEGs associated with CRC shows that a significant proportion of them are involved in the regulation of tumor cell proliferation–one of the key processes in carcinogenesis. Previous studies have shown that proteins encoded by the *LARP1*, *DGCR5*, *MTHFD1L*, and *ALOX12B* genes are associated with high proliferative and invasive potential of CRC cells. Proline dehydrogenase, encoded by the *PRODH* gene, has a dual function, in some cases exhibiting both pro- and antiproliferative functions [50,51,52]. Increased expression of *IGHG2* is characteristic of CRC [67], and mutations in the *ALOX15* gene, which is a paralog of the DEG *ALOX15B*, are associated with an increased risk of CRC [63]. However, in general the role of these genes in carcinogenesis remains only partly studied.

The functions of several genes are also associated with the pathogenesis of other types of cancer. Our results showed that the genes *CARMIL3* and *LIPH* are potentially associated with carcinogenesis processes in CMS2. The *CARMIL3* gene is associated with the development of HCC [61]. The protein encoded by *CARMIL3* is involved in the regulation of p53 stability and promotes its degradation [61]. The *LIPH* gene encodes a membrane-associated protease that catalyzes the formation of 2-acetyl lysophosphatidic acid involved in the regulation of proliferation, migration, survival of cancer cells, and angiogenesis in malignant tumors of the ovary, stomach, prostate, pancreas, and liver [102]. The study by Zhuang et al. revealed an association of *LIPH* with EMT processes, cell adhesion, and actin cytoskeleton reorganization in pancreatic cancer cells [62]. *LIPH* overexpression is also associated with reduced infiltration by CD8 + T cells and Th1 cells, as well as increased infiltration by tumor-associated macrophages, which correlates with poor prognosis for patients with pancreatic cancer [62]. In samples of CMS3, only one DEG associated with carcinogenesis was identified—*DNM1P41*. The non-coding RNA may be associated with inflammatory processes and the formation of the tumor microenvironment in HCC [68]. Thus, a number of identified DEGs for which associations with CRC pathogenesis have not yet been established may nevertheless potentially play a role in certain carcinogenic processes.

Analysis of methylation profiles and gene expression levels showed that the number of DEGs, as well as differentially methylated genes and differentially expressed HML-2, partially decreases from CMS1 to CMS3. Correlation and regression analyses also showed a negative relationship between the degree of differential methylation of sites and the degree of differential expression of HML-2 loci. Hyperexpressed HML-2 is more often located within DEGs with also increased differential expression. For example, hyperexpressed HML-2_9q34.11 is located in the gene *KYAT1* in CMS1, HML-2_10q24.2—in the gene *ABCC2* in CMS1 and CMS3, HML-2_11q12.1—in *GLYATL1* for CMSs 1–4. Hypoexpressed HML-2 in some cases is located in DEGs with decreased expression. Hypoexpressed HML-2_3q21.2 is in the gene *ENPP7P4* in CMS1, HML-2_5q33.3—in the gene *SGCD* in CMS2 and CMS3, and HML-2_3q21.2—in the gene *LINC02614* in CMS1.

Expression patterns of DEGs largely correspond to patterns of differential methylation. Thus, hypermethylated DEGs more often have decreased expression. Thus, due to hypermethylation, the expression of genes with tumor promoting properties probably decreased: *TTLL3*—in CMS1 samples, *CR1*—in CMSs 1–3, *LHFPL3*—in CMS1 and CMS2. Additionally, there is an opposite trend where DEGs with increased expression are more often hypomethylated. Hypomethylation could lead to activation of several genes, for example, ABC-transporter gene *ABCC2* in CMS1 and CMS2.

CMS1 has the highest immunogenicity and previous studies have found that immunogenic tumors are characterized by increased differential expression of *HML-2* [40]. Other studies also indicate that increased expression of mobile elements is associated with increased immune infiltration in CRC and glioblastoma [103,104]. Among the described differentially methylated DEGs containing HML-2, genes associated with immune regulation include *CD48*, *FGL1*, *KYAT1*, and *CR1*. Of these, only *CR1* is not associated with CRC according to the literature. Nevertheless, this gene participates in immune cascades potentially related to various cancers. For example, *CR1* is part of the complement system and coagulation cascade. Activation of the complement system in the tumor microenvironment may promote cell dedifferentiation, proliferation, migration, and reduce apoptosis [105]. The gene *KYAT1* encodes kynurenine aminotransferase 1. Kynurenine suppresses T cell activation and helps tumors evade immune defense mechanisms in CRC [85]. Kynurenine metabolites are involved in inflammation and immunosuppression in the microenvironment of various tumor types and promote proliferation while inhibiting apoptosis in CRC via activation of the PI3K/Akt cascade [86]. The protein encoded by *CD48* is located on the surface of antigen-presenting cells [78]. It is involved in various innate and adaptive immune responses and can function as both an inhibitory and activating receptor, which makes it important in immune regulation [77,79]. Under normal physiological conditions, fibrinogen-like protein 1 encoded by *FGL1* is secreted by hepatocytes and participates in liver lipid metabolism and other processes in the liver, blood glucose regulation [76]. This gene is also a high-affinity ligand of the inhibitory receptor LAG3, expressed on activated T cells, NK cells, and plasmacytoid dendritic cells [73,74]. Fibrinogen-like protein 1 can reduce anti-tumor immunity in the liver microenvironment by accelerating T cell exhaustion and blocking their proliferation, potentially promoting metastasis [73,76]. Comparison of the above DEGs shows that hypoexpression of *CD48*, *FGL1*, and *CR1* in CMSs 1–3 may lead to reduced activation of certain immune cascades and immune responses, including those promoting tumor development. At the same time, increased expression of *KYAT1* in CMS1 may contribute to the development of an immunosuppressive microenvironment through activation of the kynurenine pathway.

In addition, *TTLL3*, *ABCC2*, and *ZNF420* are also associated with CRC pathogenesis. *TTLL3* encodes the only tubulin-tyrosine ligase responsible for glycylation of microtubules and necessary for intestinal villi formation [81]. Elevated mRNA levels of *ABCC2* were found in CRC and colorectal adenomas [83]. The *ABCC2* gene encodes one of the ABC-transporters located in the intestine cells. Expression abnormalities of these transporters have been detected in various cancers and correlate with tumor proliferation, invasive processes, metastasis presence, and inflammation [82]. The increased expression of *ZNF420* gene, also known as APAC, is associated with poor prognosis in CRC. APAC encodes a KZNF protein that can bind p53, leading to reduced acetylation of p53. Thus, APAC can negatively regulate p53, and increased expression of this gene may inhibit apoptosis [84].

Thus, in CMS1 samples, overexpression of KYAT1 with pro-tumor functions was revealed, within which there is overexpressed HML2, as well as reduced expression of TTLL3 with anti-tumor properties. Genes with potential pro-tumor activity: CR1 is hypoexpressed in CMSs 1–3, FGL1 is hypoexpressed in CMS1 and CMS2 samples, and overexpressed in CMS4 samples. Expression of ZNF420 with pro-tumor activity is decreased in CMS1–3 samples.

The conducted analysis allowed us to assume possible changes in the activity of genes associated with the mechanisms of pathogenesis of cancer and CRC, in particular. However, the functions of a number of genes containing HML-2 or localized near them require further detailed study, including characterization of effects in relevant model cell systems.

## 4. Materials and Methods

### 4.1. Clinical Samples

The study involves analysis of data on tumor tissue and corresponding adjacent non-cancerous tissue samples obtained from 63 patients with histologically confirmed CRC. Clinical samples were collected at the Federal State Budgetary Institution “N.N. Blokhin National Medical Research Center of Oncology” of the Ministry of Health of the Russian Federation, Moscow, Russia. All biological samples were gathered as part of a study approved by the local ethics committee on 30 October 2020, with informed consent obtained from each patient.

### 4.2. RNA Sequencing and Data Analysis

Total RNA isolation from fresh frozen tissues was performed manually using the RNeasy Plus Universal Mini Kit (Qiagen, Hilden, Germany) according to the manufacturer’s instructions. Tissue homogenization was performed using a Bioprep-24 homogenizer (Hangzhou Allsheng Instruments Co., Ltd., Hangzhou, China). DNAse I treatment was perform using the RNase-Free DNase Set (Qiagen, Germany) according to the manufacturer’s recommendations. The isolated RNA was eluted in 50 μL of RNase-free water. The concentration of isolated RNA was determined using the Qubit RNA HS Assay kit on a Quantus fluorimeter (Promega, Madison, WI, USA), RNA purity was determined using a NanoDrop 8000 spectrophotometer (Thermo Fisher Scientific, Waltham, MA, USA). The RNA integrity number (RIN) was assessed using a 4200 TapeStation automated electrophoresis system (Agilent Technologies, Santa Clara, CA, USA) and the RNA ScreenTape Assay reagent kit (Agilent Technologies, Santa Clara, CA, USA).

Transcriptome libraries were manually prepared using the Illumina TruSeq Stranded Total RNA reagent kit (Illumina, San Diego, CA, USA), following the manufacturer’s instructions (Document #1000000040499 v00, Illumina, USA). IDT-ILMN TruSeq RNA UD indexes were used to prevent cross-contamination between samples. Libraries were generated in two replicates for each sample. Library concentration was measured with an Infinite F Nano Plus reader. Library size distribution was determined using the Agilent D1000 Reagent Kit on an Agilent 4200 TapeStation (Agilent Technologies, USA). Automatic pooling was performed using a Tecan Freedom EVO platform (Tecan, Männedorf, Switzerland). Quality control of the pool was performed using the Agilent HS D1000 Screen Tape Reagent Kit on the Agilent 4200 TapeStation. Sequencing was performed using an Illumina NovaSeq 6000 platform, with a 200-cycle kit of S2 reagents (Illumina, USA), with 2 × 100 bp paired-end reads for 100 million reads per sample.

RNA-seq data were processed using the nf-core RNAseq pipeline (version 3.12.0) [44] with the following software versions: Cutadapt (version 3.4) for adapter trimming [106], Samtools (version 1.17) [107], STAR (version 2.7.9a) for aligning reads to the GRCh38 reference genome [108], and Salmon (version 1.10.1) for transcript quantification [107]. The quality control criteria were as follows: M Reads Mapped (Samtools) ≥ 33 M, % Mapped (Samtools) ≥ 90%, M aligned (STAR) ≥ 30 M, M aligned (Salmon) ≥ 20 M, % Mt_rRNA biotype counts ≤ 15%, % protein coding biotype counts ≥ 35%, % exon reads (RSeQC) ≥ 35%.

### 4.3. DNA Methylation Data Acquisition and Processing

Microarray genotyping of DNA samples was performed using Illumina Infinium MethylationEPIC (Illumina, USA). Samples were prepared according to Illumina protocol, using automated station Tecan Freedom EVO. Microarray scanning was performed on Illumina Iscan system with automated loading system Autoloader 2.x. Samples with at least 95% detected CpG were included in the analysis.

Methylation data obtained using the Illumina Infinium MethylationEPIC v1.0 and v2.0 (850 K) platforms were analyzed in R using the ChAMP package (version 2.21.1) [109]. To merge data from different chip versions, a custom script was employed, considering common CpG sites.

In the initial step, samples were filtered based on detection *p*-value (<0.01). Subsequently, the following probe categories were excluded:

- Sites with fewer than 3 probes in ≥5% of the samples;
- Probes located on sex chromosomes;
- Regions overlapping with known SNPs [110].

Data normalization was performed using the SWAN algorithm to minimize technical variations between Infinium I and II probes. Batch effect correction was conducted using the Combat method [111]. Methylation levels for each CpG site were expressed as β-values (the ratio of methylated signal intensity to the total signal intensity).

For gene-level analysis, the Illumina manifest was used: CpG sites located in promoter regions (TSS200 zone) were aggregated by averaging β-values. This approach resulted in a methylation matrix where each gene is represented by the average promoter value. The methodology is analogous to that described in the study [43], which focuses on integrating multi-omics data.

### 4.4. RNA-Seq Data Analysis

#### 4.4.1. Classification of Samples by CMS Signatures

To determine the molecular subtypes (CMSs) of colorectal cancer tumors, we used the CMScaller tool (version 0.99.2) [112]. This software enables RNA-seq-based classification of samples into one of the four established CMSs: CMS1, CMS2, CMS3, and CMS4. Raw gene expression values served as input data. The model was trained on a dataset containing samples already annotated for CMS, which ensured high classification accuracy and reproducibility. Probabilities of belonging to each subtype were calculated for each sample, and the most probable subtype was selected for downstream analysis. The obtained results were used to check molecular classification against clinical characteristics and assess its prognostic relevance.

#### 4.4.2. Differential Gene Expression Analysis by CMS

We used Salmon version 1.17 [24] to estimate transcript abundances based on raw sequencing data (fastq files) for gene expression quantification from RNA-seq data. The resulting quantification files in quant.sf format were used as input for DESeq2 version 1.46.0 [113]. Prior to differential expression analysis with DESeq2, the quant.sf files generated by Salmon were imported into R and combined into a single count matrix using the tximport package [114], where transcripts were mapped to genes using a GTF annotation file. Based on the combined count matrix, a DESeqDataSetFromMatrix object was created, containing sample and experimental condition information. Library size normalization factors were estimated using the estimateSizeFactors function from the DESeq2 package [113]. Low-expressed genes were filtered out during post-normalization processing by excluding genes with fewer than 5 reads in at least two samples.

Differential gene expression was determined by pairwise comparison of tumor tissue samples with matched normal tissue samples from the same patients. Statistical significance of differential expression was defined using an adjusted *p*-value threshold (Benjamini–Hochberg method, padj) ≤ 0.05 and logarithm of the relative expression values ratio (log_2_FC) threshold ≥ 1 or ≤−1 [113]. Differential expression analysis was performed separately for each CMS.

Result visualization included volcano plots (ggplot2) [115], heatmaps (pheatmap) [116], and Venn diagrams (matplotlib_venn) [117]. Volcano plots were used to assess the distribution of log_2_FC and padj values, heatmaps were used to identify sample clusters, and Venn diagrams were used to analyze shared and unique differentially expressed genes among CMSs.

#### 4.4.3. HML-2 Expression Analysis

HERV-K expression was quantified using three bioinformatics tools: GeneTEFlow (version 2020) [29], the TECount (version 2.2.3) module from TEtranscripts [28], and Telescope (version 1.0.3) [27], which apply different algorithms for analyzing high-throughput sequencing data and quantifying retrotransposon expression.

For the analysis with TECount (version 2.2.3) and Telescope (version 1.0.3), reference GTF annotation files including all mobile genetic elements were used, allowing for accurate locus-specific expression quantification. GeneTEFlow (version 2020), in turn, employs an internal mobile element database based on UCSC data [118], enabling simultaneous assessment of both gene and transposable element expression.

To evaluate the agreement between results obtained with different tools, statistical analyses were performed, including calculation of Cohen’s kappa coefficient [119], Pearson correlation coefficient, and R^2^ [120].

For Cohen’s kappa calculation, we used the following approach: log-transformed expression fold changes (log_2_FC) were converted into categorical variables (e.g., upregulated, downregulated, or not significant) for each pair of tools. Cohen’s kappa was then calculated using the standard formula, which accounts for agreement beyond chance:

(1) $$k=\;\frac{P_{0}-P_{e}}{1-P_{e}},$$

where *P*_0_ is the observed agreement and *P_e_* is the expected agreement by chance.

To assess differential expression of HERV-K mobile genetic elements, we used quantification files generated by three independent tools: TECount (TEtranscripts version 2.2.3), GeneTEFlow (version 2020), and Telescope (version 1.0.3). To improve quantification accuracy, gene and retrotransposon annotations were both provided to each tool, facilitating more precise resolution of ambiguously mapped reads. The analysis focused specifically on the HML-2 subgroup of the HERV-K family. Results from TECount, GeneTEFlow, and Telescope were filtered to retain only the reads corresponding to HML-2.

For each tool, HML-2 quantification results were imported into R in the form of count matrices. Differential expression analysis followed the same workflow described above for gene-level expression.

To annotate genomic loci containing HML-2 sequences, we used the Homo_sapiens.GRCh38.110.gtf annotation file from Ensembl (version 110). The list of HML-2 loci and their coordinates (chromosome, start, and end) was obtained from the Ensembl retrotransposon database.

An R script (version 4.3.1) was developed for this analysis. It employed the rtracklayer package [121] for importing and exporting genomic annotations, and the GenomicRanges package [122] for handling genomic intervals. Using the subsetByOverlaps() function from GenomicRanges, annotations from the GTF file overlapping the target loci were selected. The results were formatted into tables using as.data.frame(), and the tables for all loci were merged using bind_rows() from the dplyr package [123]. The final table, containing all annotations overlapping HML-2 loci, was saved as a CSV file.

### 4.5. Association of HML-2 Loci Expression with Genes Within Loci

The association between retrotransposon expression and the expression of genes located in adjacent loci was assessed using a custom Python (version 3.11.4) script (version 3.11.4). The input data included the genomic coordinates of retrotransposons and the human genome annotation file (GTF, GRCh38.110). The implemented algorithm evaluates the overlap between retrotransposon coordinates and annotated genes to determine their intragenic localization.

### 4.6. Association of HML-2 Loci Expression with Nearby Genes

To analyze the relationship between retrotransposon locus expression and the expression of nearby genes, flanking regions of 50,000 base pairs were defined on each side of the retrotransposons. The choice of this interval was based on preliminary analysis of gene distribution: the highest number of genes was observed within this distance, while further extension of the range did not result in a substantial increase in gene count.

### 4.7. Systems Biology Analysis

GO analysis and GSEA enrichment analysis were performed for genes containing HML-2 insertions and for genes located near retrotransposons. The analysis was based on a ranked list of 112 genes for which the presence of HML-2 was associated with altered expression (either upregulation or downregulation) in tumor samples compared to normal tissue. For these genes, functional ontology analysis and intersection with known molecular pathways were conducted. Subsequently, similar analyses were performed using a gene list ranked by expression changes for all genes located in proximity to retrotransposons.

### 4.8. Comparative Analysis of DNA Methylation and Gene Expression Within HERV-K Containing Genes

To analyze the relationship between DNA methylation levels and the expression of genes located near HML-2 loci, a custom Python (version 3.11.4) script was developed. The script performed the following steps: (1) identification of genes containing HML-2 insertions; (2) annotation of methylation sites by selecting CpG sites located within ±3000 base pairs from the start and end positions of each gene; (3) promoter region verification using data from the Illumina manifest to determine whether the methylation sites were located within promoter regions. Methylation sites were filtered based on the Benjamini–Hochberg adjusted *p*-value threshold (padj < 0.05) and classified as either hypo- or hypermethylated using a cutoff of |log_2_FC| > 0.1. The analysis considered all CpG sites located within genes and within 3000 base pairs of gene boundaries to capture promoter regions. For the analysis of methylation sites near or within HML-2 loci, a window of 10,000 base pairs was used. The selection of these parameters was based on published data regarding the genomic location of human promoters (±2000–3000 base pairs from the transcription start site).

### 4.9. Filtering of CpG Site-HML-2 Locus Pairs and CpG Site-Gene Pairs

The analysis was performed separately for each of the four CMSs. Only these CpG sites were included in the analysis for which the following condition was met:

(2) $${log}_{2}FoldChange<\;-0.1,$$

Or

(3) $${log}_{2}FoldChange>0.1.$$

Differential methylation for the selected sites should be statistically significant (*p*-value < 0.05).

HML-2 loci and genes were filtered by the absolute value of the logarithm of the ratio of relative expression:

(4) $${log}_{2}FoldChange<\;-1,$$

or

(5) $${log}_{2}FoldChange>1,$$

with a corresponding *p*-value below 0.05. Differential expression of HML-2 loci in this study was determined using three bioinformatics tools: GeneTEFlow, TECount and Telescope. Only HML-2 loci with differential expression detected by the telescope tool and confirmed by at least one of the other two tools were included in the analysis.

### 4.10. Correlation Analysis

The correlation test with Spearman correlation coefficient calculation was applied to the filtered pairs of differential methylation of CpG sites and differential expression of HML-2 loci values, as well as to the pairs of differential methylation of CpG sites and differential expression of genes values. Spearman correlation coefficient was chosen due to the small sample size and the absence of a pronounced linear relationship between the variables.

### 4.11. Regression Analysis for HML-2 Loci Differential Expression

A linear regression model was built:

(6) $$Y_{i}=\;\beta_{0}+\;\beta_{1}\;X_{1i}+\beta_{2}\;X_{2i}+\beta_{3}X_{1i}X_{2i}+\varepsilon_{i},$$

where $Y_{i}$ denotes value of logarithm of the ratio of a HML-2 locus expression in tumor relative to the healthy tissue (log_2_FoldChange) in the site-locus pair *i*; $X_{1i}$—logarithm of the ratio of a CpG site methylation in tumor relative to the healthy tissue (log_2_FoldChange) in the site-locus pair *i*; $X_{2i}$—a dichotomous variable that takes the value of 0 if the HML-2 locus and the CpG site in pair *i* are located on opposite DNA chains, and 1 if they are located on the same chain; $\varepsilon_{i}$ is the error term. $\varepsilon_{i}$ is assumed to be independent and distributed normally with zero mean. The term $\beta_{3}X_{1i}X_{2i}$ reflects the suggestion that the effect of differential methylation of a CpG site on locus expression may differ depending on whether the HML-2 locus and the CpG site are located on the same or different chains. Sensitivity analysis of the model was performed using the R package “sensemakr” (version 0.1.6).

### 4.12. Regression Analysis for Differential Expression of Genes, Which Include HML-2 Loci

A linear regression model was built:

(7) $$Y_{i}=\;\beta_{0}+\;\beta_{1}X_{1i}+\beta_{2}X_{2i}+\beta_{3}X_{3i}+\beta_{4}X_{1i}X_{2i}+\beta_{5}X_{1i}X_{3i}+\varepsilon_{i},$$

where $Y_{i}$ denotes value of logarithm of the ratio of a gene expression in tumor relative to the healthy tissue (log_2_FoldChange) in the site-gene pair *i*; $X_{1i}$—logarithm of the ratio of a CpG site methylation in tumor relative to the healthy tissue (log_2_FoldChange) in the site-gene pair *i*, $X_{2i}$—a dichotomous variable that takes the value of 0 if the gene and the CpG site in pair *i* are located on opposite DNA chains, and 1 if they are located on the same chain. $X_{3i}$—a dichotomous variable that takes the value of 1 if the CpG site in pair *i* is located in the promoter of a gene, and 0 if not. $\varepsilon_{i}$ is the error term. $\varepsilon_{i}$ is assumed to be independent and distributed normally with zero mean. The term $\beta_{4}X_{1i}X_{2i}$ reflects the suggestion that the effect of differential methylation of a CpG site on gene expression may differ depending on whether the gene and the CpG site are located on the same or different chains. The term $\beta_{5}X_{1i}X_{3i}$ reflects the possible influence of the location of the CpG site in the promoter on the relationship between differential methylation of CpG site and differential gene expression. Sensitivity analysis of the model was performed using the R package “sensemakr” (version 0.1.6).

### 4.13. Estimation of Immune Cell Fractions in Tumor Tissues

To analyze the representation of different cell populations, the xCell tool was used [124]. Gene expression data normalized to TPM (Transcripts Per Million) were used as input for xCell, which estimates cell type enrichment based on gene expression levels. The output of the tool is a cell type enrichment matrix indicating the relative abundance of various immune cell types across the selected subgroup. xCell provides enrichment scores rather than percentages and is designed for comparing samples rather than directly quantifying cell types.

### 4.14. Reverse Transcription and Real-Time PCR

The reverse transcription reaction was performed using the RNAscribe RT kit (Biolabmix, Novosibirsk, Russia) according to the manufacturer’s protocol, 750 ng of total RNA sample was used for each sample. The following primer pairs were used to amplify HERV-K (HML-2) sequences: GAG_fw: 5′-TAAAAATGGGCAACCATTGTCG-3′; GAG_rev: 5′-GTTGTTGTCCCTGAAAACCC-3′; ENV_fw: 5′-CATATTAATCCTTGTGTGCCTG-3′; ENV_rev: 5′-GACAAAACCGCCATCGTCATC-3′; the following primers were used for expression normalization: RPL32_H_F: 5′-tctccttctcggcatcatgg-3′ and RPL32_H_R: 5′-cgaaccctgttgtcaatgcc-3′. Amplification was carried out on CFX96 (Bio-Rad), the expression was assessed using the ΔΔCt method.

## Figures and Tables

**Table 1:** Clinical characteristics of 60 patients with colorectal cancer classified by CMS.

CMS	CMS1	CMS2	CMS3	CMS4
Total	7	22	12	19
I	2	3	0	0
II	2	11	2	13
III	3	6	10	3
IV	0	2	0	3
Median age	67	66	69	65
MSS	1	14	9	12
MSI.H	6	7	3	4
Female	4	9	3	10
Male	3	13	9	9

**Table 2:** Correlation between differential methylation of CpG sites and differential expression of genes containing the HML-2 locus and located within 3000 bp of the CG site.

	Rho	*p*-Value
CMS1	0.301	0.080
CMS2	0.187	0.015
CMS3	0.328	<0.001
CMS4	−0.650	0.160

**Table 3:** Correlation between differential methylation of CpG sites and differential expression of HML-2 loci located within 10,000 bp of each other.

	Rho	*p*-Value
CMS1	−0.271	<0.0001
CMS2	−0.237	0.001
CMS3	−0.375	<0.0001
CMS4	−0.218	0.6

## Data Availability

Data available on request in order to protect patients’ personal data.

## Supplementary Materials

The following supporting information can be downloaded at: https://www.mdpi.com/article/10.3390/epigenomes10010011/s1, References [125,126,127,128,129,130,131,132,133,134,135,136,137,138,139,140,141] cited in the supplementary text under Figure S3.

## Abbreviations

The following abbreviations are used in this manuscript:

CRC	Colorectal cancer
CMSs	Consensus molecular subtypes
HERV	Human endogenous retroviruses
MMR	Mismatch repair
TME	Tumor microenvironment

## References

[1] Bhandari A., Woodhouse M., Gupta S. (2017). Colorectal Cancer is A Leading Cause of Cancer Incidence and Mortality among Adults Younger than 50 Years in the USA: A Seer-Based Analysis with Comparison to Other Young-Onset Cancers. J. Investig. Med..

[2] Morgan E., Arnold M., Gini A., Lorenzoni V., Cabasag C.J., Laversanne M., Vignat J., Ferlay J., Murphy N., Bray F. (2023). Global burden of colorectal cancer in 2020 and 2040: Incidence and mortality estimates from GLOBOCAN. Gut.

[3] Biller L.H., Schrag D. (2021). Diagnosis and treatment of metastatic colorectal cancer: A review. JAMA.

[4] Dunne P.D., Arends M.J. (2024). Molecular pathological classification of colorectal cancer—An update. Virchows Arch..

[5] Guinney J., Dienstmann R., Wang X., Reyniès A., Schlicker A., Soneson C., Marisa L., Roepman P., Nyamundanda G., Angelino P. (2015). The Consensus Molecular Subtypes of Colorectal Cancer. Nat. Med..

[6] Luo Q., Quan Y., Liu W., Wu Z., Qiu W., Liang W., Yang P., Huang Q., Li G., Wei L. (2024). Seed and Soil: Consensus Molecular Subgroups (CMS) and Tumor Microenvironment Features Between Primary Lesions and Metastases of Different Organ Sites in Colorectal Cancer. Cancer Manag. Res..

[7] Gallois C., Pernot S., Zaanan A., Taieb J. (2018). Colorectal Cancer: Why Does Side Matter?. Drugs.

[8] Hashimoto M., Kojima Y., Sakamoto T., Ozato Y., Nakano Y., Abe T., Hosoda K., Saito H., Higuchi S., Hisamatsu Y. (2024). Spatial and single-cell colocalisation analysis reveals MDK-mediated immunosuppressive environment with regulatory T cells in colorectal carcinogenesis. eBioMedicine.

[9] Karpinski P., Rossowska J., Sasiadek M.M. (2017). Immunological landscape of consensus clusters in colorectal cancer. Oncotarget.

[10] Joanito I., Wirapati P., Zhao N., Nawaz Z., Yeo G., Lee F., Eng C.L.P., Macalinao D.C., Kahraman M., Srinivasan H. (2022). Single-cell and bulk transcriptome sequencing identifies two epithelial tumor cell states and refines the consensus molecular classification of colorectal cancer. Nat. Genet..

[11] Dang Q., Zuo L., Hu X., Zhou Z., Chen S., Liu S., Ba Y., Zuo A., Xu H., Weng S. (2024). Molecular subtypes of colorectal cancer in the era of precision oncotherapy: Current inspirations and future challenges. Cancer Med..

[12] Bannert N., Kurth R. (2006). The evolutionary dynamics of human endogenous retroviral families. Annu. Rev. Genom. Hum. Genet..

[13] Seifarth W., Frank O., Zeilfelder U., Spiess B., Greenwood A.D., Hehlmann R., Leib-Mösch C. (2005). Comprehensive analysis of human endogenous retrovirus transcriptional activity in human tissues with a retrovirus-specific microarray. J. Virol..

[14] Dervan E., Bhattacharyya D.D., McAuliffe J.D., Khan F.H., Glynn S.A. (2021). Ancient Adversary—HERV-K (HML-2) in Cancer. Front. Oncol..

[15] Marchi E., Kanapin A., Magiorkinis G., Belshaw R. (2014). Unfixed endogenous retroviral insertions in the human population. J. Virol..

[16] Jang H.S., Shah N.M., Du A.Y., Dailey Z.Z., Pehrsson E.C., Godoy P.M., Zhang D., Li D., Xing X., Kim S. (2019). Transposable elements drive widespread expression of oncogenes in human cancers. Nat. Genet..

[17] Steiner M.C., Marston J.L., Iñiguez L.P., Bendall M.L., Chiappinelli K.B., Nixon D.F., Crandall K.A. (2021). Locus-specific characterization of human endogenous retrovirus expression in prostate, breast, and colon cancers. Cancer Res..

[18] Attig J., Pape J., Doglio L., Kazachenka A., Ottina E., Young G.R., Enfield K.S., Aramburu I.V., Ng K.W., Faulkner N. (2023). Human endogenous retrovirus onco-exaptation counters cancer cell senescence through calbindin. J. Clin. Investig..

[19] Li W., Lin L., Malhorta R., Yang L., Acharya R., Poss M. (2019). A computational framework to assess genome-wide distribution of polymorphic human endogenous retrovirus-k in human populations. PLoS Comput. Biol..

[20] Gardner E.J., Lam V., Harris D., Chuang N.T., Scott E.C., Pittard W.S., Mills R.E., Devine S.E., 1000 Genomes Project Consortium (2017). The mobile element locator tool (MELT): Population-scale mobile element discovery and biology. Genome Res..

[21] Keane T.M., Wong K., Adams D.J. (2013). RetroSeq: Transposable element discovery from next-generation sequencing data. Bioinformatics.

[22] Santander C.G., Gambron P., Marchi E., Karamitros T., Katzourakis A., Magiorkinis G. (2017). STEAK: A specific tool for transposable elements and retrovirus detection in high-throughput sequencing data. Virus Evol..

[23] Lanciano S., Cristofari G. (2020). Measuring and interpreting transposable element expression. Nat. Rev. Genet..

[24] Patro R., Duggal G., Love M.I., Irizarry R.A., Kingsford C. (2017). Salmon provides fast and bias-aware quantification of transcript expression. Nat. Methods.

[25] Liao Y., Smyth G.K., Shi W. (2014). FeatureCounts: An efficient general purpose program for assigning sequence reads to genomic features. Bioinformatics.

[26] Anders S., Huber W. (2010). Differential expression analysis for sequence count data. Genome Biol..

[27] Bendall M.L., Mulder M., Iñiguez L.P., Lecanda-Sánchez A., Pérez-Losada M., Ostrowski M.A., Jones R.B., Mulder L.C.F., Reyes-Terán G., Crandall K.A. (2019). Telescope: Characterization of the retrotranscriptome by accurate estimation of transposable element expression. PLoS Comput. Biol..

[28] Jin Y., Tam O.H., Paniagua E., Hammel M. (2015). TEtranscripts: A package for including transposable elements in differential expression analysis of RNA-seq datasets. Bioinformatics.

[29] Liu X., Bienkowska J.R., Zhong W. (2020). GeneTEFlow: A Nextflow-based pipeline for analysing gene and transposable elements expression from RNA-Seq data. PLoS ONE.

[30] Li W., Lee M.-H., Henderson L., Tyagi R., Bachani M., Steiner J., Campanac E., Hoffman D.A., Geldern G., Johnson K. (2015). Human endogenous retrovirus-K contributes to motor neuron disease. Sci. Transl. Med..

[31] Bowles H., Kabiljo R., Khleifat A.A., Jones A., Quinn J.P., Dobson R.J.B., Swanson C.M., Al-Chalabi A., Iacoangeli A. (2022). An assessment of bioinformatics tools for the detection of human endogenous retroviral insertions in short-read genome sequencing data. Front. Bioinform..

[32] Ou S., Su W., Liao Y., Chougule K., Agda J.R.A., Hellinga A.J., Lugo C.S.B., Elliott T.A., Ware D., Peterson T. (2019). Benchmarking transposable element annotation methods for creation of a streamlined, comprehensive pipeline. Genome Biol..

[33] Kang Q., Guo X., Li T., Yang C., Han J., Jia L., Liu Y., Wang X., Zhang B., Li J. (2023). Identification of differentially expressed HERV-K(HML-2) loci in colorectal cancer. Front. Microbiol..

[34] Díaz-Carballo D., Acikelli A.H., Klein J., Jastrow H., Dammann P., Wyganowski T., Guemues C., Gustmann S., Bardenheuer W., Malak S. (2015). Therapeutic potential of antiviral drugs targeting chemorefractory colorectal adenocarcinoma cells overexpressing endogenous retroviral elements. J. Exp. Clin. Cancer Res..

[35] Yu C., Lei X., Chen F., Mao S., Lv L., Liu H., Hu X., Wang R., Shen L., Zhang N. (2022). ARID1A loss derepresses a group of human endogenous retrovirus-H loci to modulate BRD4-dependent transcription. Nat. Commun..

[36] Ko E.-J., Ock M.-S., Choi Y.-H., Iovanna J.L., Mun S., Han K., Kim H.-S., Cha H.-J. (2021). Human Endogenous Retrovirus (HERV)-K env Gene Knockout Affects Tumorigenic Characteristics of nupr1 Gene in DLD-1 Colorectal Cancer Cells. Int. J. Mol. Sci..

[37] Golkaram M., Salmans M.L., Kaplan S., Vijayaraghavan R., Martins M., Khan N., Garbutt C., Wise A., Yao J., Casimiro S. (2021). HERVs establish a distinct molecular subtype in stage II/III colorectal cancer with poor outcome. Npj Genom. Med..

[38] Chuong E.B., Elde N.C., Feschotte C. (2016). Regulatory evolution of innate immunity through co-option of endogenous retroviruses. Science.

[39] Natoli M., Gallon J., Lu H., Amgheib A., Pinato D.J., Mauri F.A., Marafioti T., Akarca A.U., Ullmo I., Ip J. (2021). Transcriptional analysis of multiple ovarian cancer cohorts reveals prognostic and immunomodulatory consequences of ERV expression. J. Immunother. Cancer.

[40] Topham J.T., Titmuss E., Pleasance E.D., Williamson L.M., Karasinska J.M., Culibrk L., Lee M.K.C., Mendis S., Denroche R.E., Jang G.-H. (2020). Endogenous retrovirus transcript levels are associated with immunogenic signatures in multiple metastatic cancer types. Mol. Cancer Ther..

[41] Ye X., Skokos D. (2020). Endogenous retroviral proteins provide an immunodominant but not requisite antigen in a murine immunotherapy tumor model. Oncoimmunology.

[42] Dolci M., Favero C., Tarantini L., Villani S., Bregni M., Signorini L., Valle A.D., Crivelli F., D’Alessandro S., Ferrante P. (2020). Human endogenous retroviruses env gene expression and long terminal repeat methylation in colorectal cancer patients. Med. Microbiol. Immunol..

[43] Ivancevic A., Simpson D.M., Joyner O.M., Bagby S.M., Nguyen L.L., Bitler B.G., Pitts T.M., Chuong E.B. (2024). Endogenous retroviruses mediate transcriptional rewiring in response to oncogenic signaling in colorectal cancer. Sci. Adv..

[44] nf-core/rnaseq: Nf-core/rnaseq v3.18.0-Lithium Lynx. https://zenodo.org/records/14537300.

[45] Gonzalez-Hernandez M.J., Cavalcoli J.D., Sartor M.A., Contreras-Galindo R., Meng F., Dai M., Dube D., Saha A.K., Gitlin S.D., Omenn G.S. (2014). Regulation of the Human Endogenous Retrovirus K (HML-2) Transcriptome by the HIV-1 Tat Protein. J. Virol..

[46] Mueller T., Hantsch C., Volkmer I., Staege M.S. (2018). Differentiation-dependent regulation of human endogenous retrovirus K sequences and neighboring genes in germ cell tumor cells. Front. Microbiol..

[47] Gal A., Balicza P., Weaver D., Naghdi S., Joseph S.K., Várnai P., Gyuris T., Horváth A., Nagy L., Seifert E.L. (2017). MSTO 1 is a cytoplasmic pro-mitochondrial fusion protein. EMBO Mol. Med..

[48] Russ E., Mikhalkevich N., Iordanskiy S. (2023). Expression of Human Endogenous Retrovirus Group K (HERV-K) HML-2 Correlates with Immune Activation of Macrophages and Type I Interferon Response. Microbiol. Spectr..

[49] Ng K.W., Boumelha J., Enfield K.S.S., Almagro J., Cha H., Pich O., Karasaki T., Moore D.A., Salgado R., Sivakumar M. (2023). Antibodies against endogenous retroviruses promote lung cancer immunotherapy. Nature.

[50] Donald S.P., Sun X.Y., Hu C.A., Yu J., Mei J.M., Valle D., Phang J.M. (2001). Proline oxidase. encoded by p53-induced gene-6. catalyzes the generation of proline-dependent reactive oxygen species. Cancer Res..

[51] Pandhare J., Donald S.P., Cooper S.K., Phang J.M. (2009). Regulation and function of proline oxidase under nutrient stress. J. Cell. Biochem..

[52] Olivares O., Mayers J.R., Gouirand V., Torrence M.E., Gicquel T., Borge L., Lac S., Roques J., Lavaut M.-N., Berthezène P. (2017). Collagen-derived proline promotes pancreatic ductal adenocarcinoma cell survival under nutrient limited conditions. Nat. Commun..

[53] Huang H., Yang X., Chen J., Fu J., Chen C., Wen J., Mo Q. (2019). lncRNA DGCR5 inhibits the proliferation of colorectal cancer cells by downregulating miR-21. Oncol. Lett..

[54] Li S., Jin T., Zhang J., Lou H., Yang B., Li Y., Chen C., Zhang Y. (2012). Polymorphisms of TREH. IL4R and CCDC26 genes associated with risk of glioma. Cancer Epidemiol..

[55] Yang B., Heng L., Du S., Yang H., Jin T., Lang H., Li S. (2015). Association between RTEL1. PHLDB1. and TREH polymorphisms and glioblastoma risk: A case-control study. Med. Sci. Monit..

[56] Ohta S., Bukowski-Wills J.-C., Sanchez-Pulido L., Alves F.L., Wood L., Chen Z.A., Platani M., Fischer L., Hudson D.F., Ponting C.P. (2010). The Protein Composition of Mitotic Chromosomes Determined Using Multiclassifier Combinatorial Proteomics. Cell.

[57] Ye L., Lin S.-T., Mi Y.-S., Liu Y., Ma Y., Sun H.-M., Peng Z.-H., Fan J.-W. (2016). Overexpression of LARP1 predicts poor prognosis of colorectal cancer and is expected to be a potential therapeutic target. Tumor Biol..

[58] Kim J.Y., Liu K., Zhou S., Tillison K., Wu Y., Smas C.M. (2008). Assessment of fat-specific protein 27 in the adipocyte lineage suggests a dual role for FSP27 in adipocyte metabolism and cell death. Am. J. Physiol. Endocrinol. Metab..

[59] Oh S.J. (2018). System-Wide Expression and Function of Olfactory Receptors in Mammals. Genom. Inform..

[60] Bisgin A., Meng W.-J., Adell G., Sun X.-F. (2019). Interaction of CD200 Overexpression on tumor cells with cd200r1 overexpression on stromal cells: An escape from the host immune response in rectal cancer patients. J. Oncol..

[61] Ge Y., Xiao B., Zhao R., Li B., Yang S., He K.F., Gu H.J., Zuo S. (2024). CARMIL1 regulates liver cancer cell proliferation by activating the ERK/mTOR pathway through the TRIM27/p53 axis. Int. Immunopharmacol..

[62] Zhuang H., Chen X., Wang Y., Huang S., Chen B., Zhang C., Hou B. (2022). Identification of LIPH as an unfavorable biomarkers correlated with immune suppression or evasion in pancreatic cancer based on RNA-seq. Cancer Immunol. Immunother..

[63] Habermann N., Ulrich C.M., Lundgreen A., Makar K.W., Poole E.M., Caan B., Kulmacz R., Whitton J., Galbraith R., Potter J.D. (2013). PTGS1, PTGS2, ALOX5, ALOX12, ALOX15, and FLAP SNPs: Interaction with fatty acids in colon cancer and rectal cancer. Genes Nutr..

[64] Liu Z., Li L., Li X., Hua M., Sun H., Zhang S. (2021). Prediction and prognostic significance of ALOX12B and PACSIN1 expression in gastric cancer by genome-wide RNA expression and methylation analysis. J. Gastrointest. Oncol..

[65] Cui J., Wang L., Zhong W., Chen Z., Chen J., Yang H., Liu G. (2020). Identification and validation of methylation-driven genes prognostic signature for recurrence of laryngeal squamous cell carcinoma by integrated bioinformatics analysis. Cancer Cell Int..

[66] Sang Q., Li B., Kuang Y., Wang X., Zhang Z., Chen B., Wu L., Lyu Q., Fy Y., Yan Z. (2018). Homozygous Mutations in WEE2 Cause Fertilization Failure and Female Infertility. Am. J. Hum. Genet..

[67] Boncheva V.B., Linnebacher M., Kdimati S., Draper H., Orchard L., Mills K.I., O’Sullivan G., Tangney M., Guinn B.-A. (2022). Identification of the Antigens Recognised by Colorectal Cancer Patients Using Sera from Patients Who Exhibit a Crohn’s-like Lymphoid Reaction. Biomolecules.

[68] Shi M., Zhu S., Sun L., Hu J., Li H., Dai W., Song N., Li M., Wu Y., Xu D. (2023). Transcriptome-Wide Dynamics of m7G-Related LncRNAs during the Progression from HBV Infection to Hepatocellular Carcinoma. Front. Biosci..

[69] Logar C.M., Chen W., Schmitt H., Yu C.Y., Birmingham D.J. (2004). A human CR1-like transcript containing sequence for a binding protein for iC4 is expressed in hematopoietic and fetal lymphoid tissue. Mol. Immunol..

[70] Tiihonen J., Koskuvi M., Lähteenvuo M., Trontti K., Ojansuu I., Vaurio O., Cannon T.D., Lönnqvist J., Therman S., Suvisaari J. (2021). Molecular signaling pathways underlying schizophrenia. Schizophr. Res..

[71] Dunkelberger J.R., Song W.C. (2010). Complement and its role in innate and adaptive immune responses. Cell Res..

[72] Agarwal S., Behring M., Hale K., Diffalha S.A., Wang K., Manne U., Varambally S. (2019). MTHFD1L. A Folate Cycle Enzyme, Is Involved in Progression of Colorectal Cancer. Transl. Oncol..

[73] Wang J., Sanmamed M.F., Datar I., Su T.T., Ji L., Sun J., Chen L., Chen Y., Zhu G., Yin W. (2019). Fibrinogen-like Protein 1 Is a Major Immune Inhibitory Ligand of LAG-3. Cell.

[74] Woo S.R., Turnis M.E., Goldberg M.V., Bankoti J., Selby M., Nirschl C.J., Bettini M.L., Gravano D.M., Vogel P., Liu C.L. (2012). Immune inhibitory molecules LAG-3 and PD-1 synergistically regulate T-cell function to promote tumoral immune escape. Cancer Res..

[75] Li J.J., Wang J.-H., Tian T., Liu J., Zheng Y.-Q., Mo H.-Y., Sheng H., Chen Y.-X., Wu Q.-N., Han Y. (2023). The liver microenvironment orchestrates FGL1-mediated immune escape and progression of metastatic colorectal cancer. Nat. Commun..

[76] Qian W., Zhao M., Wang R., Li H. (2021). Fibrinogen-like protein 1 (FGL1): The next immune checkpoint target. J. Hematol. Oncol..

[77] Pahima H., Puzzovio P.G., Levi-Schaffer F. (2019). 2B4 and CD48: A powerful couple of the immune system. Clin. Immunol..

[78] Zou C., Zhu C., Guan G., Guo Q., Liu T., Shen S., Yan Z., Xu X., Lin Z., Chen L. (2019). CD48 is a key molecule of immunomodulation affecting prognosis in glioma. Onco Targets Ther..

[79] McArdel S.L., Terhorst C., Sharpe A.H. (2016). Roles of CD48 in regulating immunity and tolerance. Clin. Immunol..

[80] He M., Yu J., Chen S., Mi H. (2023). A Systematic Immune and Prognostic Analysis of CD48 Interaction with Tumor Microenvironment in Pan-Cancer. Int. J. Gen. Med..

[81] Rocha C., Papon L., Cacheux W., Sousa P.M., Lascano V., Tort O., Giordano T., Vacher S., Lemmers B., Mariani P. (2014). Tubulin glycylases are required for primary cilia. control of cell proliferation and tumor development in colon. EMBO J..

[82] Muriithi W., Macharia L.W., Heming C.P., Echevarria J.L., Nyachieo A., Filho P.N., Neto V.M. (2020). ABC transporters and the hallmarks of cancer: Roles in cancer aggressiveness beyond multidrug resistance. Cancer Biol. Med..

[83] Andersen V., Vogel L.K., Kopp T.I., Sæbø M., Nonboe A.W., Hamfjord J., Kure E.H., Vogel U. (2015). High ABCC2 and low ABCG2 gene expression are early events in the colorectal adenoma-carcinoma sequence. PLoS ONE.

[84] Olcina M.M., Leszczynska K.B., Senra J.M., Isa N.F., Harada H., Hammond E.M. (2016). H3K9me3 facilitates hypoxia-induced p53-dependent apoptosis through repression of APAK. Oncogene.

[85] Wang Z., Yin M., Zhou R., Li M., Peng M., Wang Z. (2025). Kynurenine promotes the immune escape of colorectal cancer cells via NAT10-mediated ac4C acetylation of PD-L1. Clinics.

[86] Bishnupuri K.S., Alvarado D.M., Khouri A.N., Shabsovich M., Chen B., Dieckgraefe B.K., Ciorba M.A. (2019). IDO1 and kynurenine pathway metabolites activate PI3K-Akt signaling in the neoplastic colon epithelium to promote cancer cell proliferation and inhibit apoptosis. Cancer Res..

[87] He J.R., Xi J., Ren Z.-F., Qin H., Zhang Y., Zeng Y.-Z., Mo H.-Y., Jia W.-H. (2012). Complement receptor 1 expression in peripheral blood mononuclear cells and the association with clinicopathological features and prognosis of nasopharyngeal carcinoma. Asian Pac. Organ. Cancer Prev..

[88] Hu Y., Hu Q., Li Y., Lu L., Xiang Z., Yin Z., Kabelitz D., Wu Y. (2023). γδ T cells: Origin and fate, subsets, diseases and immunotherapy. Signal Transduct. Target. Ther..

[89] Angelova P.R., Kasymov V., Christie I., Sheikhbahaei S., Turovsky E., Marina N., Korsak A., Zwicker J., Teschemacher A.G., Ackland G.L. (2015). Functional Oxygen Sensitivity of Astrocytes. J. Neurosci..

[90] Kinker G.S., Vitiello G.A.F., Ferreira W.A.S., Chaves A.S., Lima V.C.C., Medina T.S. (2021). B Cell Orchestration of Anti-tumor Immune Responses: A Matter of Cell Localization and Communication. Front. Cell Dev. Biol..

[91] Pan Y., Yu Y., Wang X., Zhang T. (2020). Tumor-Associated Macrophages in Tumor Immunity. Front. Immunol..

[92] Saetang J., Tedasen A., Sangkhathat S., Sangkaew N., Dokduang S., Prompat N., Taraporn S., Graidist P. (2022). The attenuation effect of low piperine Piper nigrum extract on doxorubicin-induced toxicity of blood chemical and immunological properties in mammary tumour rats. Pharm. Biol..

[93] Luo H., Duan M., Kong L., He L., Chen Y., Wang Z., Tang X. (2021). The Regulatory Mechanism of 2-Acetyl-1-Pyrroline Biosynthesis in Fragrant Rice (*Oryza sativa* L.) Under Different Soil Moisture Contents. Front. Plant Sci..

[94] Coënon L., Geindreau M., Ghiringhelli F., Villalba M., Bruchard M. (2024). Natural Killer cells at the frontline in the fight against cancer. Cell Death Dis..

[95] Becht E., Reyniès A., Giraldo N.A., Pilati C., Buttard B., Lacroix L., Selves J., Sautès-Fridman C., Laurent-Puig P., Fridman W.H. (2016). Immune and stromal classification of Colorectal cancer is associated with molecular subtypes and relevant for precision immunotherapy. Clin. Cancer Res..

[96] Raskov H., Orhan A., Christensen J.P., Gögenur I. (2021). Cytotoxic CD8+ T cells in cancer and cancer immunotherapy. Br. J. Cancer.

[97] Wylie B., Macri C., Mintern J.D., Waithman J. (2019). Dendritic cells and cancer: From biology to therapeutic intervention. Cancers.

[98] Del Prete A., Salvi V., Soriani A., Laffranchi M., Sozio F., Bosisio D., Sozzani S. (2023). Dendritic cell subsets in cancer immunity and tumor antigen sensing. Cell. Mol. Immunol..

[99] Hugo H., Ackland M.L., Blick T., Lawrence M.G., Clements J.A., Williams E.D., Thompson E.W. (2007). Epithelial—Mesenchymal and mesenchymal—Epithelial transitions in carcinoma progression. J. Cell. Physiol..

[100] Goerner-Potvin P., Bourque G. (2018). Computational tools to unmask transposable elements. Nat. Rev. Genet..

[101] Holicek P., Guilbaud E., Klapp V., Truxova I., Spisek R., Galluzzi L., Fucikova J. (2024). Type I interferon and cancer. Immunol. Rev..

[102] Lin M.E., Herr D.R., Chun J. (2010). Lysophosphatidic acid (LPA) receptors: Signaling properties and disease relevance. Prostaglandins Other Lipid Mediat..

[103] Kong Y., Rose C.M., Cass A.A., Williams A.G., Darwish M., Lianoglou S., Haverty P.M., Tong A.-J., Blanchette C., Albert M.L. (2019). Transposable element expression in tumors is associated with immune infiltration and increased antigenicity. Nat. Commun..

[104] Zhu X., Fang H., Gladysz K., Barbour J.A., Wong J.W.H. (2021). Overexpression of transposable elements is associated with immune evasion and poor outcome in colorectal cancer. Eur. J. Cancer.

[105] Afshar-Kharghan V. (2017). The role of the complement system in cancer. J. Clin. Investig..

[106] Martin M. (2011). Cutadapt removes adapter sequences from high-throughput sequencing reads. EMBnet J..

[107] Danecek P., Bonfield J.K., Liddle J., Marshall J., Ohan V., Pollard M.O., Whitwham A., Keane T., McCarthy S.A., Davies R.M. (2021). Twelve years of SAMtools and BCFtools. Gigascience.

[108] Dobin A., Davies C.A., Schlesinger F., Drenkow J., Zaleski C., Jha S., Batut P., Chaisson M., Gingeras T.R. (2013). STAR: Ultrafast universal RNA-seq aligner. Bioinformatics.

[109] Tian Y., Morris T.J., Webster A.P., Yang Z., Beck S., Feber A., Teschendorff A.E. (2017). ChAMP: Updated methylation analysis pipeline for Illumina BeadChips. Bioinformatics.

[110] Zhou W., Laird P.W., Shen H. (2017). Comprehensive characterization, annotation and innovative use of Infinium DNA methylation BeadChip probes. Nucleic Acids Res..

[111] Wang M., Huang J., Liu Y., Ma L., Potash J.B., Han S. (2017). COMBAT: A Combined Association Test for Genes Using Summary Statistics. Genetics.

[112] Eide P.W., Bruun J., Lothe R.A., Sveen A. (2017). CMScaller: An R package for consensus molecular subtyping of colorectal cancer pre-clinical models. Sci. Rep..

[113] Love M.I., Huber W., Anders S. (2014). Moderated estimation of fold change and dispersion for RNA-seq data with DESeq2. Genome Biol..

[114] Soneson C., Love M.I., Robinson M.D. (2016). Differential analyses for RNA-seq: Transcript-level estimates improve gene-level inferences. F1000Research.

[115] R Package ggplot2.

[116] R: Draw a Heat Map [Electronic Resource]. https://search.r-project.org/R/refmans/stats/html/heatmap.html.

[117] GitHub—Konstantint/Matplotlib-Venn: Area-Weighted Venn-Diagrams for Python/Matplotlib [Electronic Resource]. https://github.com/konstantint/matplotlib-venn?tab=readme-ov-file.

[118] Karolchik D., Hinrichs A.S., Furey T.S., Roskin K.M., Sugnet C.W., Haussler D., Kent W.J. (2004). The UCSC table browser data retrieval tool. Nucleic Acids Res..

[119] Cohen J. (1960). A Coefficient of Agreement for Nominal Scales. Educ. Psychol. Meas..

[120] Pearson K. (1896). Mathematical Contributions to the Theory of Evolution. III. Regression, Heredity, and Panmixia. Philos. Trans. A.

[121] Lawrence M., Gentleman R., Carey V. (2009). rtracklayer: An R package for interfacing with genome browsers. Bioinformatics.

[122] Lawrence M., Huber W., Pagès H., Aboyoun P., Carlson M., Gentleman R., Morgan M.T., Carey V.J. (2013). Software for Computing and Annotating Genomic Ranges. PLoS Comput. Biol..

[123] Bioconductor—Organism.dplyr [Electronic Resource]. https://bioconductor.org/packages/release/bioc/html/Organism.dplyr.html.

[124] Aran D., Hu Z., Butte A.J. (2017). xCell: Digitally portraying the tissue cellular heterogeneity landscape. Genome Biol..

[125] Zhao Y., Huang Z., Gao L., Ma H., Chang R. (2024). Osteopontin/SPP1: A potential mediator between immune cells and vascular calcification. Front. Immunol..

[126] Lester S.N., Li K. (2014). Toll-like receptors in antiviral innate immunity. J. Mol. Biol..

[127] Lancioni C.L., Li Q., Thomas J.J., Ding X.D., Thiel B., Drage M.G., Pecora N.D., Ziady A.G., Shank S., Harding C.V. (2011). Mycobacterium tuberculosis lipoproteins directly regulate human memory CD4+ T cell activation via toll-like receptors 1 and 2. Infect. Immun..

[128] Georgel P., Jiang Z., Kunz S., Janssen E., Mols J., Hoebe K., Bahram S., Oldstone M.B.A., Beutler B. (2007). Vesicular stomatitis virus glycoprotein G activates a specific antiviral Toll-like receptor 4-dependent pathway. Virology.

[129] Hayashi F., Smith K.D., Ozinsky A., Hawn T.R., Yi E.C., Goodlett D.R., Eng J.K., Akira S., Underhill D.M., Aderem A. (2001). The innate immune response to bacterial flagellin is mediated by Toll-like receptor 5. Nature.

[130] Cox D.G., Pontes C., Guino E., Navarro M., Osorio A., Canzian F., Moreno V., Bellvitge Colorectal Cancer Study Group (2004). Polymorphisms in prostaglandin synthase 2/cyclooxygenase 2 (PTGS2/COX2) and risk of colorectal cancer. Br. J. Cancer.

[131] Yang J., McNeish B., Butterfield C., Moses M.A. (2013). Lipocalin 2 is a novel regulator of angiogenesis in human breast cancer. FASEB J..

[132] Li P., Wang J., Zou Y., Sun Z., Zhang Z., Geng Z., Xu W., Wang D. (2019). Interaction of Hsp90AA1 with phospholipids stabilizes membranes under stress conditions. Biochim. Biophys. Acta Biomembr..

[133] Riyapa D., Buddhisa S., Korbsrisate S., Cuccui J., Wren B.W., Stevens M.P., Ato M., Lertmemongkolchai G. (2012). Neutrophil extracellular traps exhibit antibacterial activity against Burkholderia pseudomallei and are influenced by bacterial and host factors. Infect. Immun..

[134] Jenne C.N., Wong C.H.Y., Zemp F.J., McDonald B., Rahman M.M., Forsyth P.A., McFadden G., Kubes P. (2013). Neutrophils recruited to sites of infection protect from virus challenge by releasing neutrophil extracellular traps. Cell Host Microbe.

[135] Thanarajasingam U., Jensen M.A., Dorschner J.M., Muskardin T.W., Ghodke-Puranik Y., Purmalek M., Eliopoulos E., Zervou M.I., Goulielmos G.N., Howard M. (2017). A Novel ELANE Mutation Associated With Inflammatory Arthritis. Defective NETosis, and Recurrent Parvovirus Infection. Arthritis Rheumatol..

[136] Liu C., Yalavarthi S., Tambralli A., Zeng L., Rysenga C.E., Alizadeh N., Hudgins L., Liang W., NaveenKumar S.K., Shi H. (2023). Inhibition of neutrophil extracellular trap formation alleviates vascular dysfunction in type 1 diabetic mice. Sci. Adv..

[137] Saitoh T., Komano J., Saitoh Y., Misawa T., Takahama M., Kozaki T., Uehata T., Iwasaki H., Omori H., Yamaoka S. (2012). Neutrophil extracellular traps mediate a host defense response to human immunodeficiency virus-1. Cell Host Microbe.

[138] Wang H., Kim S.J., Lei Y., Wang S., Wang H., Huang H., Zhang H., Tsung A. (2024). Neutrophil extracellular traps in homeostasis and disease. Signal Transduct. Target. Ther..

[139] Wang R., Dong X., Zhang X., Liao J., Cui W., Li W. (2025). Exploring viral sensor combined with epigenetics and tumor immunity: New perspectives in cancer therapy. Int. J. Biol. Sci..

[140] Park Y.J., Oanh N.T.K., Heo J., Kim S.-G., Lee H.-S., Lee H., Lee J.-H., Kang H.C., Lim W., Yoo Y.-S. (2020). Dual targeting of RIG-I and MAVS by MARCH5 mitochondria ubiquitin ligase in innate immunity. Cell Signal..

[141] Arimoto K.-I., Takahashi H., Hishiki T., Konishi H., Fujita T., Shimotohno K. (2007). Negative regulation of the RIG-I signaling by the ubiquitin ligase RNF125. Proc. Natl. Acad. Sci. USA.

