# Supplementary material for: Analysis of HERV-K (HML2) Expression in Colorectal Cancer Samples
Source: Epigenomes. 2026 Feb 12;10(1):11. doi: 10.3390/epigenomes10010011 (PMC12922068; doi:10.3390/epigenomes10010011)
Supplement: Supplementary file 1 [file epigenomes-10-00011-s001.zip › Supplementary Figures.pdf]

Supplementary figures

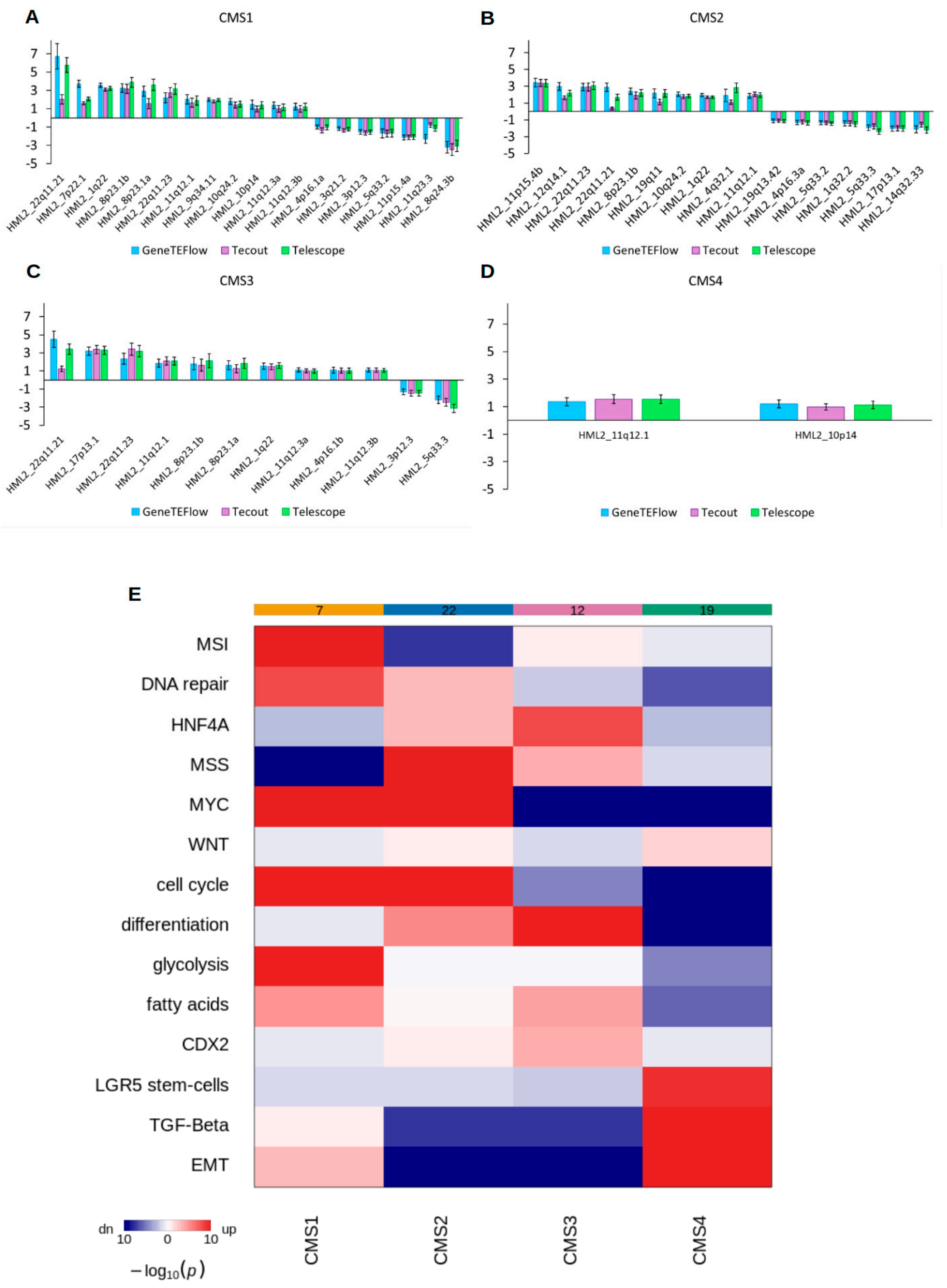

Figure S1. (A – D) Distribution of differential expression levels of HERV-K loci in CRC samples of CMSs 1–4, identified using three tools: GeneTEFlow, TEcount, and Telescope. (A) Bar chart showing the distribution of HERV-K expression levels in CRC CMS1 samples; (B) Bar chart showing the distribution of HERV-K expression levels in CRC CMS2 samples; (C) Bar chart showing the distribution of HERV-K expression levels in CRC CMS3 samples; (D) Bar chart showing the distribution of HERV-K expression levels in CRC CMS4 samples. Error bars

represent standard deviation values; (E) – Heatmap showing the results of mRNA gene set enrichment analysis, confirming the enrichment of known features in each CMS group. Red and blue indicate relative upregulation and downregulation, respectively (dn: downregulation; up: upregulation), and the intensity of color reflects increasing statistical significance. Pathways are described in the original publication about CMScaller [51]

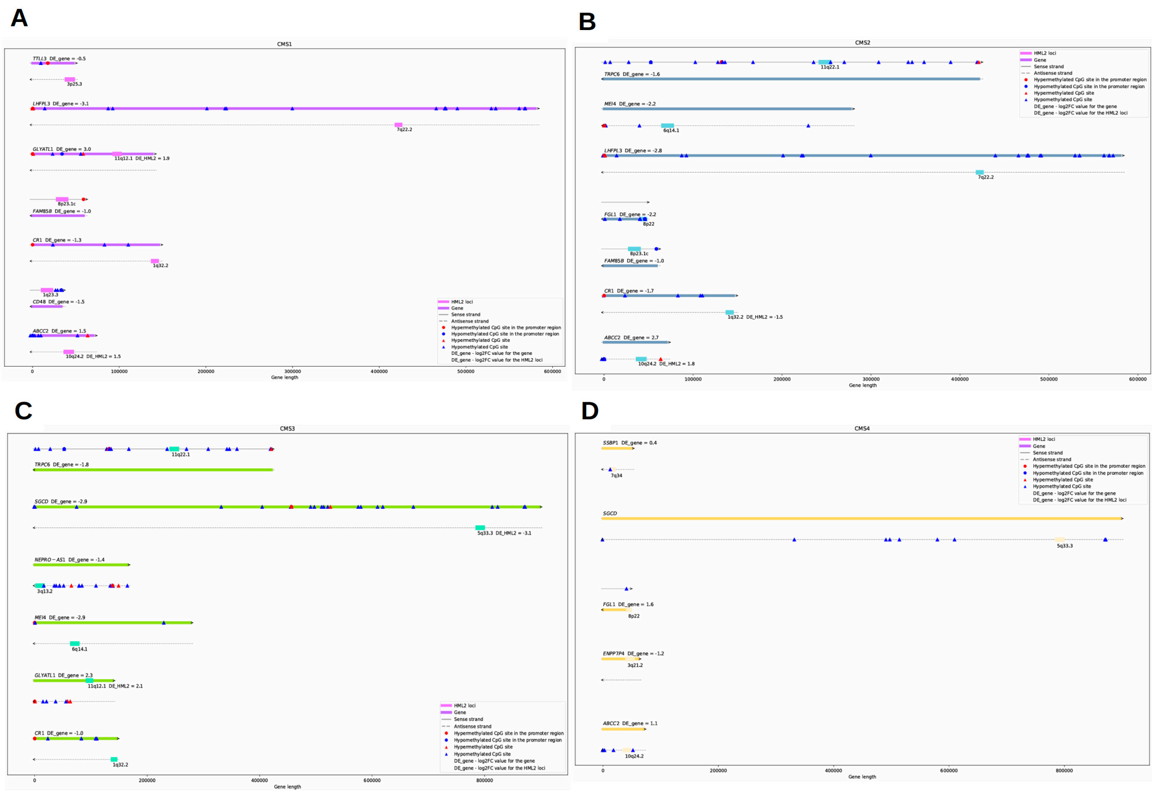

Figure S2. (A) Schematic representation of genes for CMS1 with indication of the sense and antisense strands. The figure shows genes with HML-2 loci, located on both the sense and antisense strands. Hypo- and hypermethylation in the promoter regions are indicated by dots, while in the gene body, they are represented by triangles. Differential expression of genes and HML-2 loci, which passed statistical significance, is also shown; (B) Schematic representation of genes for CMS2 with indication of the sense and antisense strands. The figure shows genes with HML-2 loci, located on both the sense and antisense strands. Hypo- and hypermethylation in the promoter regions are indicated by dots, while in the gene body, they are represented by triangles. Differential expression of genes and HML-2 loci, which passed statistical significance, is also shown; (C) Schematic representation of genes for CMS3 with indication of the sense and antisense strands. The figure shows genes with HML-2 loci, located on both the sense and antisense strands. Hypo- and hypermethylation in the promoter regions are indicated by dots, while in the gene body, they are represented by triangles. Differential expression of genes and HML-2 loci, which passed statistical significance, is also shown; (D) Schematic representation of genes for CMS3 with indication of the sense and antisense strands. The figure shows genes with HML-2 loci, located on both the sense and antisense strands. Hypo- and hypermethylation in the promoter regions are indicated by dots, while in the gene body, they are represented by triangles. Differential expression of genes and HML-2 loci, which passed statistical significance, is also shown

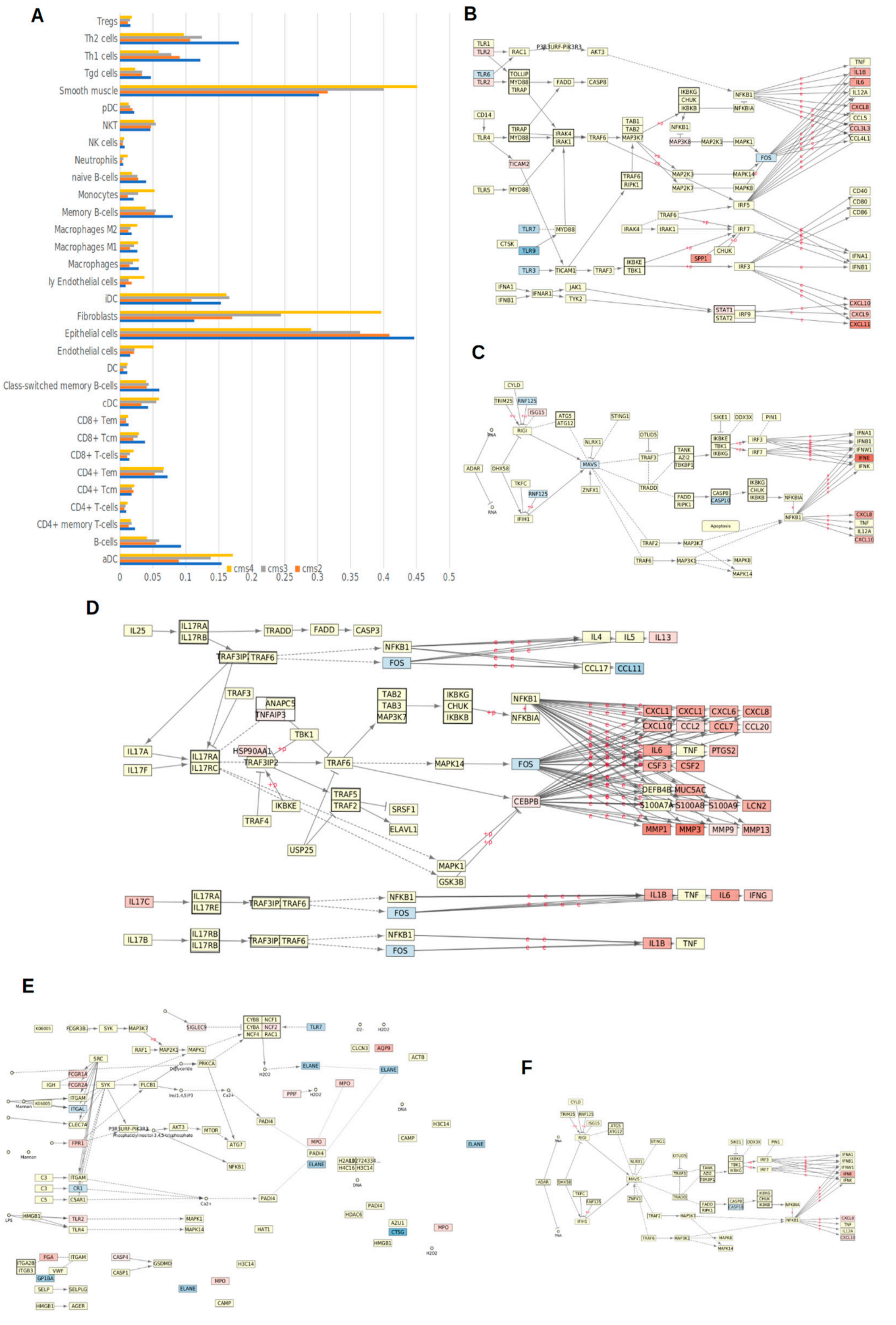

Figure S3. (A) Average proportions of cell populations in colorectal cancer tumor samples calculated for each molecular subtype (CMS1, CMS2, CMS3, CMS4); (B) Diagram of the Toll-like receptor (TLR) signaling cascade indicating differentially expressed genes in the CMS1. Genes significantly upregulated in CMS1 (log2FC > 1) are highlighted in red, while genes with downregulated expression (log2FC < -1) are shown in blue; (C) Signaling cascade of RIG-I-like receptors (RLR) indicating differentially expressed genes in CMS1; (D) Diagram of the interleukin-17 (IL-17) signaling cascade indicating differentially expressed genes in CMS1; (E) Signaling cascade of neutrophil extracellular trap (NET) formation with indication of differentially expressed genes in CMS1; (F) Signaling cascade of RIG-I-like receptors (RLR) indicating differentially expressed genes in the CMS4. Genes

significantly upregulated in CMS1 (log2FC ≥ 1) are highlighted in red, while genes with downregulated expression (log2FC ≤ -1) are shown in blue

For each CMS, we identified several signaling cascades, associated with the implementation of various immune responses, according to literature data. Differences in differential gene expression are observed in these cascades depending on the CMS.

Thus, hyperexpression of some cascade participants is registered for the CMS1 subtype and, to a somewhat lesser extent, for CMS4. For the other two subtypes, CMS2 and CMS3, fewer DEGs are observed in these cascades, or these genes are hypoexpressed. This is consistent with previous studies, as increased immune cell infiltration is characteristic of CMS1 and CMS4 [9].

When considering immune cascades separately, a significant number of DEGs are observed in CMS1 that correspond to an increased log fold change in relative expression values (|log2FC| ≥ 1). This applies to proinflammatory cytokines and chemokines, participants of the Toll-like receptor signaling pathway (TLR cascade) (Figure S3B), such as *IL1B* (log2FC=4.39), *IL6* (log2FC=4.98), *CXCL8* (log2FC=4.5), *CCL3L3* (log2FC=3.14), *CXCL9* (log2FC=3.14), *CXCL10* (log2FC=3.52), *CXCL11* (log2FC=6.26). Increased expression of osteopontin *SPP1* (log2FC=5.47), which participates in the regulation of immune response and inflammation, is also observed in this cascade [119].

The differential expression of the above genes in the TLR cascade indicates activation of the innate immune response. However, hypoexpression of most receptors recognizing viral nucleic acids (*TLR3*, *TLR6*, *TLR7*, and *TLR9*) is also observed [120]. Hyperexpression of *TLR2* (log2FC = 1.69) was also detected; it plays a role in the response to viral infection but can also be activated by bacterial components and tumor antigens [120]. Other receptors in this cascade, *TLR1*, *TLR4*, *TLR5*, are not DEGs. *TLR1* is also activated by bacterial components and can cooperate with *TLR2* to provide innate immune response upon detection of bacterial lipoproteins [121]. *TLR4* may be involved in the response to viral infection and can stimulate type 1 interferon production [122]. *TLR5* can recognize bacterial flagellin [123].

Increased log2FC is observed in the cascade's terminal effectors of the IL-17 signaling pathway (Figure S3D). High expression of cytokines and chemokines *CXCL1* (log2FC=4.75), *CXCL8* (log2FC=3.97), *CCL2* (log2FC=2.21), *CCL7* (log2FC=4.26), *IL6* (log2FC=4.98), *CSF2* (log2FC=4.82), *CSF3* (log2FC=4.21) indicates a strong proinflammatory response. Increased expression of metalloproteinases *MMP1* (log2FC=6.15), *MMP3* (log2FC=6.83), *MMP9* (log2FC=1.64), *MMP13* (log2FC=3.26) may indicate extracellular matrix degradation. Lipocalin *LCN2* (log2FC=4.4) and prostaglandin synthase *PTGS2* (log2FC=3.59) are also associated with inflammation and angiogenesis [124,125]. Additionally, a slight increase in log2FC for *TNFAIP3* (log2FC=1.02), a negative cascade regulator, and *HSP90AA1* (log2FC=1.69), a chaperone involved in stabilization and maintenance of signaling protein homeostasis, was noted [126]. However, expression of key cascade regulators does not change, and the transcription factor *FOS* (log2FC=-1.30) is hypoexpressed.

Thus, pronounced activation of several IL-17-dependent signaling pathway effectors, including proinflammatory cytokines, is observed in analyzed CMS1 samples.

Neutrophil extracellular traps (NET) formation is the body's response to pathogens, especially bacterial infections, but they also participate in inflammation and antiviral response [127,128]. We observe activation of the NET cascade (Figure S3E), evidenced by increased expression (log2FC ≥ 1) of genes associated with neutrophil activation and NET formation (*FCGR1A, FCGR2A, FPR1, MPO, FGA, AQP9, PPIF, NCF2*). However, decreased expression of elastase *ELANE* (log2FC = -2.13) and Toll-like receptor *TLR7* (log2FC = -1.75), involved in NET formation, was also registered [129–131]. A slight hyperexpression of lectin *SIGLEC9*, capable of suppressing NET formation, is observed [132].

All of the above relates to CMS1 . A similar pattern is observed for CMS4 , but log2FC values for hyperexpressed genes are lower than in CMS1. In CMS2 and CMS3, as expected, significantly fewer DEGs were found, many of which are hypoexpressed (log2FC ≤ -1). Overall, these results correspond to CMS characteristics described in previous studies [114].

The RIG-I-like receptor signaling pathway plays a key role in antiviral innate immune response, and its activation may be associated with viral mimicry [133]. In analyzed CMS1 CRC samples (Figure S3C), hyperexpression of cascade terminal effectors such as *IFNE* (log2FC=7.73), *CXCL8* (log2FC=4.5), and *CXCL10*

(log2FC=3.5) is observed, which may indicate activation of the antiviral response. Additionally, hypoexpression of ubiquitin ligase *RNF125* (log2FC=-1.25), which acts as a negative cascade regulator, was noted [134,135]. Hypoexpression of mitochondrial antiviral signaling protein *MAVS* (log2FC=-1.1), a key adaptor signaling molecule necessary for cascade activation, was also observed [134]. A similar pattern is observed for CMS4 (Figure S3F), where *IFNE* (log2FC=4.21), *CXCL8* (log2FC=2.04), and *CXCL10* (log2FC=1.69) are also hyperexpressed but to a lesser extent than in CMS1. In contrast, analysis of this cascade in CMS2 and CMS3 samples (Figure S4A-B) shows fewer DEGs in the cascades considered. Only two DEGs were noted in CMS2 (*RNF125* and *CXCL10*) and three DEGs in CMS3 (*TBKBP1*, *CXCL8*, and *CXCL10*). It should be noted that this signaling pathway did not reach statistical significance after multiple testing correction (p-value > 0.05) by GSEA results for any CMS. According to ClueGO, RIG-I-like receptor cascades are statistically significant in all subtypes except CMS4.

A

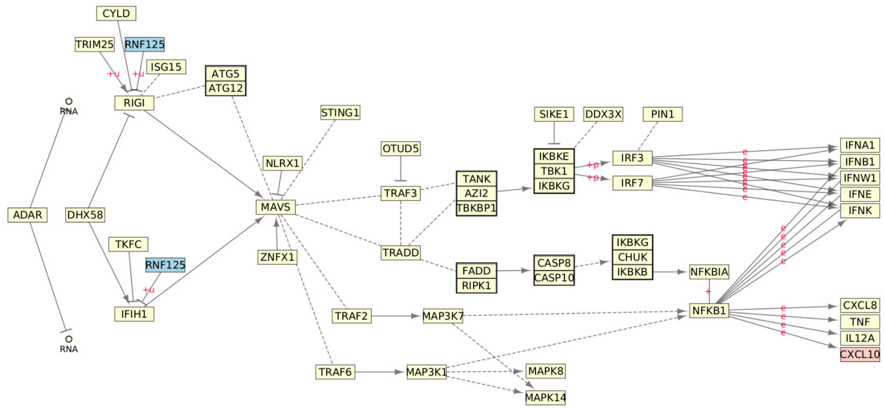

B

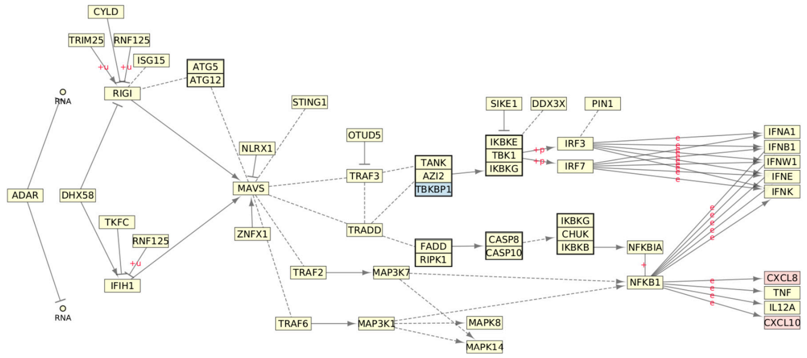

Figure S4. (A) The RIG-I-like receptor (RLR) signaling cascade with differentially expressed genes marked in CMS2; (B) The RIG-I-like receptor (RLR) signaling cascade showing differentially expressed genes in CMS3.

Genes with significantly increased expression in CMS1 (log2FC ≥ 1) are marked in red, while genes with decreased expression (log2FC ≤ -1) are marked in blue
