# Supplementary material for: Analysis of HERV-K (HML2) Expression in Colorectal Cancer Samples
Source: Epigenomes. 2026 Feb 12;10(1):11. doi: 10.3390/epigenomes10010011 (PMC12922068; doi:10.3390/epigenomes10010011)
Supplement: Supplementary file 1 [file epigenomes-10-00011-s001.zip › Supplementary tables.pdf]

Table S1 – Differential expression of genes containing HML-2 endogenous retroviral element loci in CMS1 samples. The table includes only genes with statistically significant expression changes, selected based on the adjusted p-value threshold (padj<0.05)*.

gene_name	log2FoldChange_gene	loci_name	log2FoldChange_loci_telescope	log2FoldChange_loci_geneteflow	log2FoldChange_loci_tecount
NEPRO-AS1	-0.2995	HML-2_3q13.2	0.9018	1.6597	0.5409
TTLL3	-0.5105	HML-2_3p25.3	0.2286	0.6486	0.0760
...					

* The complete table with additional parameters and statistical values is available upon request

Table S2 – Differential expression of genes containing HML-2 endogenous retroviral element loci in CMS2 samples. The table includes only genes with statistically significant expression changes, selected based on the adjusted p-value threshold (padj<0.05)*.

gene_name	log2FoldChange_gene	loci_name	log2FoldChange_loci_telescope	log2FoldChange_loci_geneteflow	log2FoldChange_loci_tecount
MEI4	-2.9077	HML-2_6q14.1	-0.1835	-0.1594	-0.0427
ENSG00000255240	2.1598	HML-2_11q12.1	2.1128	2.0869	1.8538
...					

* The complete table with additional parameters and statistical values is available upon request

Table S3 – Differential expression of genes containing HML-2 endogenous retroviral element loci in CMS3 samples. The table includes only genes with statistically significant expression changes, selected based on the adjusted p-value threshold (padj<0.05)*.

gene_name	log2FoldChange_gene	loci_name	log2FoldChange_loci_telescope	log2FoldChange_loci_geneteflow	log2FoldChange_loci_tecount
MEI4	-2.9077	HML-2_6q14.1	-0.1835	-0.1594	-0.0427
ENSG00000255240	2.1598	HML-2_11q12.1	2.1128	2.0869	1.8538
...					

* The complete table with additional parameters and statistical values is available upon request

Table S4 – Differential expression of genes containing HML-2 endogenous retroviral element loci in CMS4 samples. The table includes only genes with statistically significant expression changes, selected based on the adjusted p-value threshold (padj<0.05)*.

gene_name	log2FoldChang	loci_name	log2FoldChan	log2FoldChange	log2FoldChange

	e_gene		ge_loci_telescope	_loci_geneteflow	_loci_tecount
ENSG00000287839	0.8291	HML-2_1q22	0.8561	0.9213	0.8144
PCAT14	2.3070	HML-2_22q11.23	0.8297	2.2992	2.2574
…					

* The complete table with additional parameters and statistical values is available upon request

Table S5 – Differential expression of genes located near HML-2 endogenous retroviral element loci in CMS1 samples. Only genes with statistically significant expression changes (padj<0.05) are included*.

gene_name	log2FoldChange_gene	loci_name	log2FoldChange_loci_telescope	log2FoldChange_loci_geneteflow	log2FoldChange_loci_tecount	distance_loci_to_gene
ENSG00000255495	-0.8698	HML-2_8p23.1d	-0.3873	-0.2270	-0.3765	20181
ZNF252P-AS1	-0.9619	HML-2_8q24.3b	-3.4795	-3.0797	-3.2286	15198
…						

* The complete table with additional parameters and statistical values is available upon request

Table S6 – Differential expression of genes located near HML-2 endogenous retroviral element loci in CMS2 samples. Only genes with statistically significant expression changes (padj<0.05) are included*.

gene_name	log2FoldChange_gene	loci_name	log2FoldChange_loci_telescope	log2FoldChange_loci_geneteflow	log2FoldChange_loci_tecount	distance_loci_to_gene
ZNF732	-1.5155	HML-2_4p16.3a	-1.2428	-1.3421	-1.3280	20181
DHRS4L2	-0.4233	HML-2_14q11.2	0.6742	0.5972	0.6120	15198
…						

* The complete table with additional parameters and statistical values is available upon request

Table S7 – Differential expression of genes located near HML-2 endogenous retroviral element loci in CMS3 samples. Only genes with statistically significant expression changes (padj<0.05) are included*.

gene_id	log2FoldChange_gene	loci_name	log2FoldChange_loci_telescope	log2FoldChange_loci_geneteflow	log2FoldChange_loci_tecount	distance_loci_to_gene
ENSG00000214999	1.5697	HML-2_17p13.1	3.3681	3.2879	3.1912	15581
ENSG00000260599	-2.0720	HML-2_19p12b	-0.0252	-0.1879	0.7407	47073
…						

* The complete table with additional parameters and statistical values is available upon request

Table S8 – Differential expression of genes located near HML-2 endogenous retroviral element loci in CMS4 samples. Only genes with statistically significant expression changes (padj<0.05) are included*.

gene_name	log2FoldChange_gene	padj_gene	loci_name	log2FoldChange_loci_telescope	log2FoldChange_loci_geneteflow	distance_loci_to_gene
ENSG00000255495	-0.5704	2.26e-04	HML-2_8p23.1d	-0.3610	-0.4418	20181
ARPC4	-0.3889	2.36e-04	HML-2_3p25.3	-0.4389	-0.2286	40561
…						

* The complete table with additional parameters and statistical values is available upon request

Table S9 – Comprehensive DNA methylation and gene expression data for CpG sites located within genes containing HML-2 endogenous retroviral element loci in CMS1 samples. Only CpG sites with statistically significant methylation changes (padj<0.05) are included*.

CpG site (cg)	CpG position (cg_pos)	Methylation change (log2FC_cg)	Gene name (gene_name)	Gene expression change (log2FoldChange_gene)	HML-2 locus (loci)
cg23613225	113019398	-0.0038	NEPRO-AS1	-0.2995	HML-2_3q13.2
cg19091756	9810178	-0.0046	TTLL3	-0.5105	HML-2_3p25.3

...

* The complete table with additional parameters and statistical values is available upon request

Table S10 – Comprehensive DNA methylation and gene expression data for CpG sites located within genes containing HML-2 endogenous retroviral element loci in CMS2 samples. Only CpG sites with statistically significant methylation changes (padj<0.05) are included*.

CpG site (cg)	CpG position (cg_pos)	Methylation change (log2FC_cg)	Gene name (gene_name)	Gene expression change (log2FoldChange_gene)	HML-2 locus (loci)
cg04046323	9809883	-0.003619958	TTLL3	-0.038348177	HML-2_3p25.3
cg07925487	141738668	-0.003795704	SSBP1	0.811870547	HML-2_7q34
...					

* The complete table with additional parameters and statistical values is available upon request

Table S11 – Comprehensive DNA methylation and gene expression data for CpG sites located within genes containing HML-2 endogenous retroviral element loci in CMS3 samples. Only CpG sites with statistically significant methylation changes (padj<0.05) are included*.

CpG site (cg)	CpG position (cg_pos)	Methylation change (log2FC_cg)	Gene name (gene_name)	Gene expression change (log2FoldChange_gene)	HML-2 locus (loci)
cg04085771	42890961	-0.006507239	TTLL3	0.440245675	HML-2_6p21.1
cg00007300	24052225	-0.007279775	SSBP1		HML-2_14q11.2
...					

* The complete table with additional parameters and statistical values is available upon request

Table S12 – Comprehensive DNA methylation and gene expression data for CpG sites located within genes containing HML-2 endogenous retroviral element loci in CMS4 samples. Only CpG sites with statistically significant methylation changes (padj<0.05) are included*.

CpG site (cg)	CpG position (cg_pos)	Methylation change	Gene name (gene_name)	Gene expression	HML-2 locus (loci)

		(log2FC_cg)		change (log2FoldChange_gene)	
cg06239285	62337482	-0.00602765	ASRGL1	-0.06631916	HML-2_11q12.3a
cg14147224	141738529	-0.009111344	SSBP1	0.36506670	HML-2_7q34
...					

* The complete table with additional parameters and statistical values is available upon request

Table S13 – Results of regression analysis of the relationship between differential methylation of CG sites and differential expression of HML-2 loci located within 10.000 bp from each other.

	**Regression coefficient**		
	β_1 (95% confidence interval; p-value)	β_3 (95% confidence interval; p-value)	Robustness Value (%)
CMS1	-1.18 (-3.09:0.73; 0.22)	-1.93 (-4.92:1.05; 0.20)	8.1
CMS2	-4.07 (-7.71:-0.43; 0.03)	0.82 (-3.68:5.32; 0.72)	15.33
CMS3	-0.95 (-2.22:0.32; 0.14)	-0.86 (-2.65:0.92; 0.34)	10.14
CMS4	-0.4 (-36.21:35.41; 0.98)	-0.73 (-39.93:38.47; 0.96)	1.63

Table S14 – Results of regression analysis of the relationship between differential methylation of CG sites and differential expression of genes containing HML-2 loci and located within 3.000 bp from CG sites.

	**Regression coefficient**			
	β_1 (95% confidence interval; p-value)	β_4 (95% confidence interval; p-value)	β_5 (95% confidence interval; p-value)	Robustness Value (%)
CMS1	1.07 (-6.31:8.45; 0.11)	5.03 (-3.85:13.92; 0.26)	-1.52 (-12.31:9.27; 0.78)	5.34
CMS2	1.69 (-0.80:4.19; 0.18)	2.53 (-0.65:5.70; 0.12)	-8.44 (-13.26:-3.62; <0.0001)	10.0
CMS3	2.25 (0.19:4.31; 0.03)	2.77 (0.03:5.51; 0.048)	-8.41 (-14.72:-2.10; 0.01)	18.09

Table S15 – Results of intergroup comparison of cell populations between subtypes using the Kruskal–Wallis test.

**Cell type**	**pval**	**effsize**	**adjpval**
**B-cells**	0.5956	0	1

Cell type	pval	effsize	adjpval
**CD4+ T-cells**	0.0161	0.063	0.1742
**CD4+ Tcm**	0.8193	0	1
**CD4+ Tem**	0.3258	0.004	1
**CD4+ memory T-cells**	0.2661	0.0083	1
**CD8+ T-cells**	0.0116	0.0692	0.1494
**CD8+ Tcm**	0.0685	0.0354	0.5927
**CD8+ Tem**	0.3505	0.0024	1
**DC**	0.0009	0.1153	0.0247
**NK cells**	0.291	0.0064	1
**NKT**	0.3831	0.0005	1
**Tgd cells**	0.0864	0.0309	0.6922
**Th1 cells**	0.1254	0.0236	0.8569
**Th2 cells**	0.0959	0.0288	0.6922
**Tregs**	0.2577	0.0089	1
**aDC**	0.0008	0.1191	0.0247
**cDC**	0.0017	0.1049	0.0311
**class-switched memory B-cells**	0.5442	0	1
**endothelial cells**	0.003	0.0942	0.0489
**epithelial cells**	0.0127	0.0675	0.1494
**fibroblasts**	0.0001	0.1646	0.004
**iDC**	0.0366	0.0475	0.3399
**lymphoid endothelial cells**	0.0108	0.0706	0.1494
**macrophages**	0.0003	0.1341	0.0146
**macrophages M1**	0.0014	0.1088	0.0294
**macrophages M2**	0.0928	0.0295	0.6922
**memory B-cells**	0.4563	0	1
**monocytes**	0	0.186	0.0025
**naive B-cells**	0.7541	0	1
**neutrophils**	0.2402	0.0104	1
**pDC**	0.2148	0.0127	1
**smooth muscle**	0.0305	0.051	0.3047

Table S16 – Pairwise comparisons of cell populations between subtypes using Dunn's test with Benjamini–Yekutieli correction (adjusted p<0.05).

Cell type	CMS1 vs CMS2	CMS1 vs CMS3	CMS1 vs CMS4	CMS2 vs CMS3	CMS2 vs CMS4	CMS3 vs CMS4
**DC**	0.058	1	1	0.241	0.002	0.667
**aDC**	0.059	1	1	0.059	0.004	1
**cDC**	0.894	0.894	0.637	0.054	0.004	1
**endothelial cells**	0.003	0.29	0.853	0.302	0.003	0.561
**fibroblasts**	0.039	0.648	1	0.393	0.005	0.648
**macrophages**	0.137	1	0.642	0.137	0	0.156
**macrophages M1**	1	1	0.029	1	0	0.002
**monocytes**	1	1	0.209	1	0.004	0.209
